# Normal Hematopoietic Progenitor Subsets Have Distinct Reactive Oxygen Species, BCL2 and Cell-Cycle Profiles That Are Decoupled from Maturation in Acute Myeloid Leukemia

**DOI:** 10.1371/journal.pone.0163291

**Published:** 2016-09-26

**Authors:** Naeem Khan, Robert K. Hills, Steve Knapper, Lora Steadman, Ushna Qureshi, Jerrald L. Rector, Charlotte Bradbury, Nigel H. Russell, Paresh Vyas, Alan K. Burnett, David Grimwade, Paul S. Hole, Sylvie D. Freeman

**Affiliations:** 1 Department of Clinical Immunology, Institute of Immunology and Immunotherapy, University of Birmingham, Edgbaston, Birmingham, United Kingdom; 2 Department of Haematology, Cardiff University School of Medicine, University Hospital Wales, Cardiff, United Kingdom; 3 School of Cellular and Molecular Medicine, University of Bristol, Bristol, United Kingdom; 4 Centre for Clinical Haematology, Nottingham University Hospital NHS Trust, Nottingham, United Kingdom; 5 Weatherall Institute of Molecular Medicine, University of Oxford, Oxford, United Kingdom; 6 Department of Medical and Molecular Genetics, King’s College London School of Medicine, Guy’s & St. Thomas’ NHS Foundation Trust, London, United Kingdom; European Institute of Oncology, ITALY

## Abstract

In acute myeloid leukemia (AML) quiescence and low oxidative state, linked to BCL2 mitochondrial regulation, endow leukemic stem cells (LSC) with treatment-resistance. LSC in CD34^+^ and more mature CD34^−^ AML have heterogeneous immunophenotypes overlapping with normal stem/progenitor cells (SPC) but may be differentiated by functional markers. We therefore investigated the oxidative/reactive oxygen species (ROS) profile, its relationship with cell-cycle/BCL2 for normal SPC, and whether altered in AML and myelodysplasia (MDS). In control BM (n = 24), ROS levels were highest in granulocyte-macrophage progenitors (GMP) and CD34^−^ myeloid precursors but megakaryocyte-erythroid progenitors had equivalent levels to CD34^+^CD38^low^ immature-SPC although they were ki67^high^. BCL2 upregulation was specific to GMPs. This profile was also observed for CD34^+^SPC in MDS-without-excess-blasts (MDS-noEB, n = 12). Erythroid CD34^−^ precursors were, however, abnormally ROS-high in MDS-noEB, potentially linking oxidative stress to cell loss. In pre-treatment AML (n = 93) and MDS-with-excess-blasts (MDS-RAEB) (n = 14), immunophenotypic mature-SPC had similar ROS levels to co-existing immature-SPC. However ROS levels varied between AMLs; *Flt3*ITD^+^*/NPM1*wild-type CD34^+^SPC had higher ROS than *NPM1*mutated CD34^+^ or CD34^−^ SPC. An aberrant ki67^low^BCL2^high^ immunophenotype was observed in CD34^+^AML (most prominent in *Flt3*ITD AMLs) but also in CD34^−^ AMLs and MDS-RAEB, suggesting a shared redox/pro-survival adaptation. Some patients had BCL2 overexpression in CD34^+^ ROS-high as well as ROS-low fractions which may be indicative of poor early response to standard chemotherapy. Thus normal SPC subsets have distinct ROS, cell-cycle, BCL2 profiles that in AML /MDS-RAEB are decoupled from maturation. The combined profile of these functional properties in AML subpopulations may be relevant to differential treatment resistance.

## Introduction

Recent years have witnessed accelerated interest in the role of reactive oxygen species (ROS) in both normal hematopoietic development and leukemogenesis and in how redox modulation may impact on therapeutic strategies for acute myeloid leukemia (AML) [1,2].

ROS serve as mediators in proliferation and differentiation of hematopoietic cells, either via a paracrine mechanism [3], or by intracellular ROS production induced by cytokine receptor engagement on hematopoietic cells [4–7]. Normal hematopoietic stem cells (HSC) however require fine-tuning of low intracellular ROS levels in order to maintain quiescence, limit oxidative stress damage and sustain lifelong haematopoiesis. The metabolic adjustment of HSCs to reliance on glycolysis with resulting low ROS levels is due to the local conditions of hypoxic bone marrow (BM) niches, and also transcription/signalling pathways that alter ROS homeostasis and are critical for HSC function [8]. Gene targeting of regulatory molecules such as FoxO and Atm leads to higher ROS in HSC and loss of quiescence and self-renewal [9,10]. Conversely, manipulations that decrease ROS levels cause increased HSC quiescence and loss of effective differentiation into downstream progenitors [11]. Inefficient ROS homeostasis resulting in oxidative stress and genetic instability in HSCs and myeloid progenitors has been linked to myeloid malignancy; [12–14] for example high ROS levels have been correlated with greater DNA damage in AML with *Flt3*-internal-tandem-duplication (ITD) [15] and may explain the poor prognosis [16]. Furthermore overproduction of ROS in AML drives growth factor independent proliferation of AML blasts [17,18].

Flow cytometric analyses with fluorescent ROS-indicator dyes show that quiescent drug-resistant cancer and AML leukemic stem cells (LSC) are enriched in the ROS-low fraction of total tumour cells [2,19] similarly to normal slow-cycling HSC being enriched in the ROS-low population [20]. Unlike HSC and most cancer cells [21], AML LSC have increased dependence on oxidative phosphorylation despite maintaining protective low ROS levels [2]. This mitochondrial respiration appears to require BCL2, the overexpression of which has previously been established to have other roles in functional chemo-resistance through anti-apoptosis [22] and potentially by inducing quiescence [23]. These and other mechanisms for BCL2 dependence suggest that chemo-resistant AML LSC may be targeted by the novel BCL2 inhibitor ABT199 /venetoclax [24,25]. However identifying and tracking chemo-resistant LSCs in patients to evaluate the effectiveness of such new therapies or indeed standard treatments is problematic since xenotransplantation assays are not applicable to routine clinical practice and LSC immunophenotypic profiles are heterogeneous. LSC have been identified within the lineage-negative CD34^+^CD38^-/low^ compartment that contains HSC in normal haematopoiesis [26,27] but also in ‘more mature’ CD34^+^CD38^+^ [28] and CD34^−^ progenitor/precursor compartments [29,30]. Potentially, low intracellular ROS levels could be used as a differential functional marker of chemo-resistant leukemic stem/progenitor cells (SPC) within immunophenotypic subsets pre- and post-treatment, particularly if combined with quiescence and BCL2 expression. It remains unclear, however, how ROS levels are modulated in the various normal human progenitors downstream from normal HSC compared to their immunophenotypic leukemic counterparts in AML [28] or at the intermediate/pre-leukemic stage of MDS. In addition the relationship between ROS, BCL2 and cell-cycle status for the different normal and leukemic SPC subsets requires further clarification in order to understand the relationship between emergence of SPCs with potential chemo-resistant functional properties and maturation stage in both CD34^+^ and more mature CD34^−^ AML subtypes. We sought to investigate this, developing a novel combined flow cytometry assay to measure ROS, cell-cycle (ki67) and the anti-apoptotic marker BCL2 in blast populations from normal, MDS and AML patients.

## Methods

### Patient samples

All experiments with human clinical material were performed after receiving approval from the University of Birmingham Research Governance office and the North West—Greater Manchester East Research Ethics Committee (12/NW/0742) and were conducted according to the principles expressed in the Declaration of Helsinki. Human BM and peripheral blood (PB) specimens were residual material from clinical samples for which institutional/ethical approvals were obtained. Control samples were adult (ages 43-84yrs, median 64yrs) lymphoma patient staging BM (n = 24) with no evidence of haematological malignant cells (confirmed by morphology and flow cytometry), and umbilical cord blood (UCB) samples (n = 4). Pre-treatment BM/PB samples from AML patients (n = 93, details in S1 File and S1 Table) and BM samples from myelodysplastic syndrome (MDS)/myeloproliferative disease (MPD) patients were also studied (n = 26, age 32-89yrs, median 76yrs). All samples were between 24–36 hours old.

### Cell staining and flow cytometry

For ROS analysis, cells were labelled with 2‘-7‘-dichlorofluorescein diacetate (Life Technologies), hereafter abbreviated to DCF, and then with monoclonal antibodies (mAb) against SPC markers (details in supporting information S1 File [supplementary methods], S1 Fig, and panels in S2 Table). DCF median fluorescent intensity (MFI) values of control and diseased SPCs were standardised by dividing these values with the DCF-MFI value of lymphocytes present within each sample. CD45^int^CD117^+^ gating was performed to identify blasts. CD34^+^CD38^low^ cells were sub-divided into HSC (CD90^+^CD45RA^−^), multipotent progenitors (MPP; CD45RA^−^CD90^−^) and lymphoid-primed multipotent progenitors (LMPP; CD45RA^+^CD90^−^). CD34^+^CD38^high^ cells were sub-divided into CMP (CD45RA^−^CD123^+^), GMP (CD45RA^+^CD123^+^) and MEP (CD45RA^−^CD123^−^). For intracellular staining experiments chloro-methyl-DCF was used followed by surface and intracellular staining (ki67/BCL2 and isotype controls). For viability assays, cells were stained with Annexin-V and 7-aminoactinomycin D (7AAD). Data acquisition was performed on a BD-FACS-Canto-II flow cytometer and later analysed with FlowJo (v7.6) software.

### Colony forming unit (CFU) assays

To determine lineage potential FACS-sorted immunophenotypic subsets were seeded into methylcellulose media (Methocult, StemCell Technologies) at 100–1000 cells per dish. CFU-dishes were incubated at 37°C, 5%CO_2_ for 14-days and then scored for colonies using an inverted light microscope. Specific colony types (CFU-GEMM, CFU-GM, CFU-M, CFU-G and CFU-E) were expressed as percentage of total colony yield under each experimental condition.

### Drug sensitivity assays

Freshly isolated (by Ficoll-based density gradient centrifugation) pre-treatment AML patient cells were incubated at 37°C 5%CO_2_ with or without ABT199 (Caltag Medsystems) for 16 hours at a range of concentrations in duplicate. Sensitivity to ara-C (Sigma-Aldrich) was measured after incubation with drug for 48 hours. After washing, cells were stained for surface markers and then with Annexin-V and 7AAD. Live/dead cells were then visualized by flow cytometry with toxicity calculated using the formula: %specific apoptosis = (test-control)×100 / (100-control)

## Results

### ROS levels in control stem/progenitor cells (SPC)

**Fig 1:**
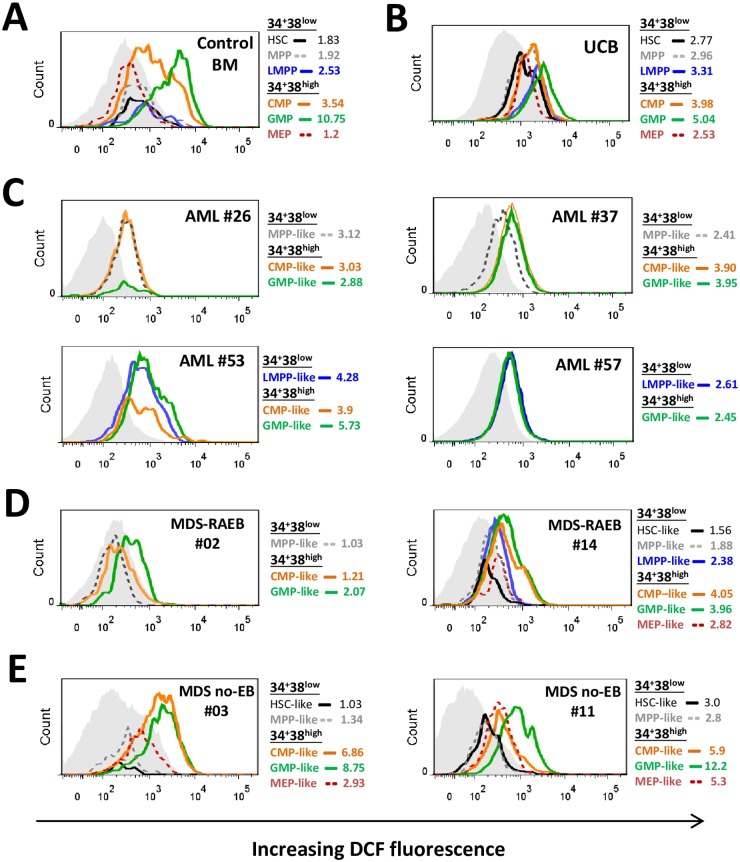
ROS levels in immunophenotypic stem/progenitor cells from representative normal control, myelodysplastic and AML bone marrows. Flow cytometry histograms show intracellular ROS levels (using DCF) in HSC/MPP/LMPP and CMP/GMP/MEP of representative control BM (A), umbilical cord blood (UCB) (B), four AML BM samples at diagnosis (C), two MDS-RAEB BM samples (D), and two MDS-no-EB BM samples (E). DCF staining with normalised mean fluorescence intensity of each of these populations is shown (using DCF staining of reference lymphocyte population shown as light grey filled histogram, while for stem/progenitor subsets staining is shown as open histograms with different coloured lines as indicated on each plot). Additional examples of DCF staining of AML and MDS samples are shown in S1F–S1H Fig.

**Fig 2:**
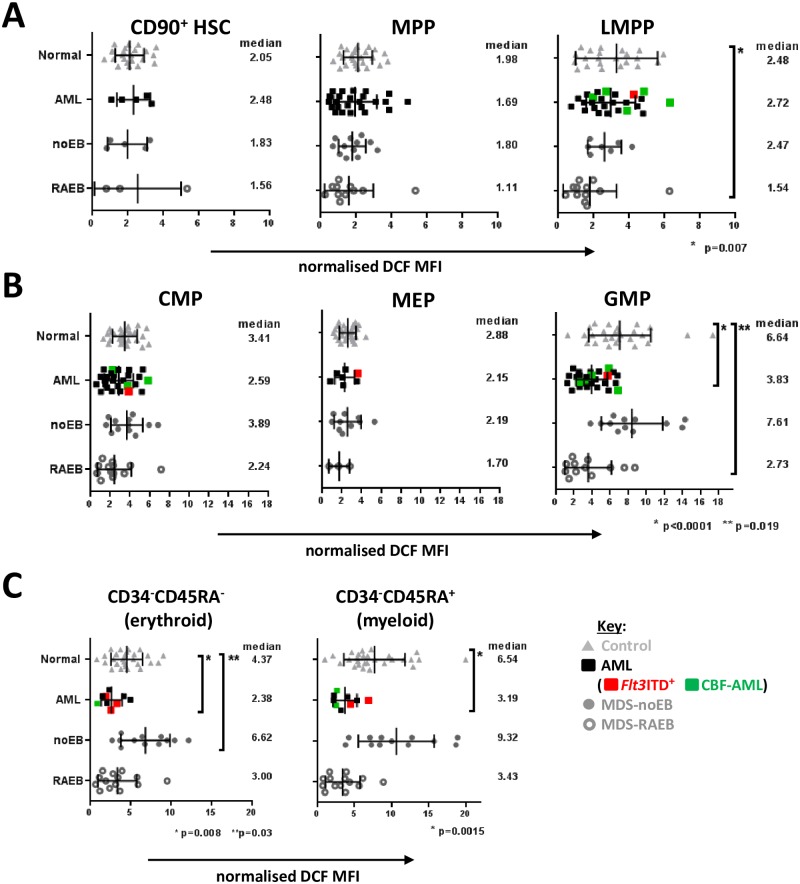
Summarised ROS levels in control, dysplastic and leukemic CD34^+^ and CD34^−^ blasts. Charts show normalised DCF MFI (relative to lymphocytes within each sample) of CD34^+^CD38^low^ subsets (A) and CD34^+^CD38^high^ subsets (B) from control BM (n = 24), and AML BM (n = 27), MDS/MPD-no-EB BM (n = 12) and MDS-RAEB BM (n = 14). *Flt3*ITD^+^ AML = red filled square. CBF-AMLs = green filled squares (see Key in figure). ROS levels in CD34^−^CD117^+^ cells were also compared between control BM and CD34^−^ AML (n = 10) and MDS patient BM samples, using the same colour scheme as above (C). CD34^−^CD117^+^ cells were subdivided into CD45RA^+^ and CD45RA^−^cells to enrich for myeloid and erythroid precursors respectively. Median expression and interquartile range is shown on each plot. P values are shown for data-sets where significant differences were observed (p<0.05, Mann Whitney test with 95% confidence intervals).

We first asked whether low ROS levels measured with the redox-sensitive probe DCF were restricted to, and could thus differentiate HSC from, other immunophenotypic normal human hematopoietic progenitor populations (S1 Fig). In control BM samples (n = 24) total CD34^+^CD38^high^ cells (fraction containing committed progenitors) had higher DCF staining than total CD34^+^CD38^low^ cells (fraction containing more immature SPC) as expected. Further analysis within these two immunophenotypic compartments (representative examples in Fig 1A) confirmed that HSC had low DCF staining (median standardised-DCF-MFI = 2.05), as did the MPP population (median 1.98). The infrequent LMPP subset (<5% of CD34^+^CD38^low^) had relatively higher DCF staining (median 2.48), compared to both HSC and MPP. Interestingly there was a differential ROS profile within the CD34^+^CD38^high^ fraction: GMP had highest DCF staining (median 6.64), followed by CMP (median 3.41) and then MEP (median 2.88) (complete summarised data in Fig 2A and 2B). The hierarchy in DCF staining with GMP>CMP>MEP and LMPP>HSC/MPP was also observed in umbilical cord blood (UCB) samples (n = 4) (example in Fig 1B), suggesting that this ROS profile is representative of normal non-malignant human SPC and not specific to the BM microenvironment.

### Lineage fate and viability of CD34^+^CD38^high^ cells with different ROS levels

**Fig 3:**
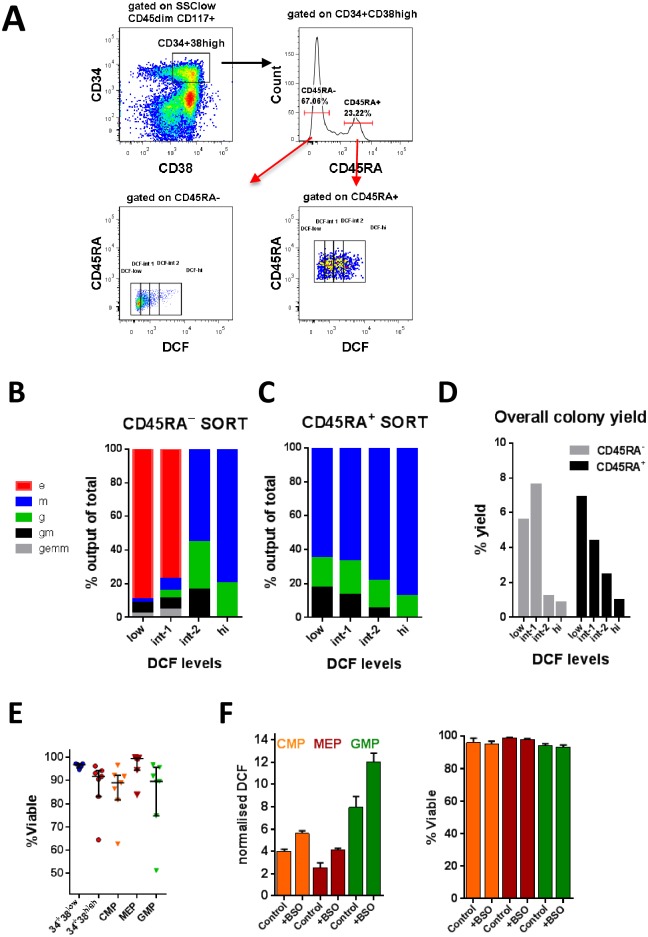
Influence of ROS levels on lineage fate and viability in normal progenitors. Sorting scheme of normal BM CD34^+^CD38^high^ cells separated into CD45RA^+^ and CD45RA^−^subsets, followed by gating into 4 different populations based on differential DCF staining (A). Sorted cells were seeded onto methylcellulose media supplemented with cytokines. After 2 week culture at 37°C in vitro, colonies were scored using an inverted light microscope and recorded as a percentage of the total colony yield for FACS-sorted control CD34^+^CD38^high^ CD45RA^−^(B) and CD34^+^CD38^high^ CD45RA^+^ (C) progenitors with different levels of DCF staining. Total colony yield as percentage of input cell number was also determined (D). Colony forming unit assay data is from three independent experiments. Colonies were scored as erythroid (e), macrophage (m), granulocyte (g), granulocyte-macrophage (gm), or granulocyte-erythroid-macrophage mixed (gemm). Viability was determined by Annexin V and 7-AAD staining of normal BM labelled with HSPC-specific mAb. Initial gating was performed on SSC^low^ CD45^int^CD117^+^ cells. The pooled viability data from 7 control BM sample are shown (E). The effects of redox modification on DCF staining and cell viability in CD34^+^CD38^high^ progenitors, is shown from two independent experiments (F). BM cells were treated overnight with the pro-oxidant BSO (100μM) or left untreated (control).

The relationship between ROS levels and immunophenotypic normal progenitors (CD34^+^CD38^high^ fraction) was further assessed by colony assays using purified progenitor subsets from control BM (n = 3). CD34^+^CD38^high^CD45RA^+^ cells (GMP-enriched) and CD34^+^CD38^high^CD45RA^−^cells (CMP/MEP-enriched), were sorted into four populations denoted DCF^low^, DCF^int1^, DCF^int2^ and DCF^high^ (Fig 3A) and assayed for colony output after 14 days culture in vitro. Results from CD45RA^−^cells showed that mixed (CFU-GEMM) and erythroid (CFU-E) potential was limited to DCF^low^/DCF^int1^ fractions and was lost in DCF^int2^/DCF^high^ fractions. Only granulocyte/macrophage colonies were generated in DCF^int2^ and DCF^high^ fractions (Fig 3B). CD45RA^+^ cells exclusively generated granulocyte (CFU-G) and macrophage (CFU-M) or mixed GM colonies (CFU-GM) with a relative reduction in CFU-GM and increase in CFU-M observed with increasing DCF (DCF^int2^/DCF^high^ fraction) (Fig 3C). Thus, higher ROS in the CD45RA^−^(CMP/MEP-enriched) compartment correlates with reduced multipotency, loss of erythroid potential and commitment towards GMP while higher ROS in the CD45RA^+^ (GMP-enriched) compartment correlates with loss of mixed GM potential and higher macrophage potential.

High intracellular ROS levels may predispose GMP to greater risk of apoptosis. In CFU assays, total colony yields were progressively lower with increasing ROS levels (Fig 3D), suggesting that higher ROS impedes cell proliferation and survival during cell culture. We evaluated cell viability in control BM cells (n = 7) (Fig 3E). Total CD34^+^CD38^low^ cells and MEP, which are both low for ROS, had the highest viability (>96.5%) while CMP and GMP, which are high in ROS, both had lower frequencies of viable Annexin-V^−^/7AAD^−^cells (86% and 84% respectively). We investigated whether SPCs with higher ROS might be more sensitive to apoptosis by further oxidative insult (such as that generated by chemotherapy) by overnight treatment with the pro-oxidant BSO. This caused increased DCF staining but did not alter the number of apoptotic cells within CMP/GMP subsets (Fig 3F and 3G), suggesting that these cells have a relative protection against rapid induction of apoptosis from higher ROS levels.

### Altered ROS levels in AML and MDS CD34^+^ cells

We next evaluated the heterogeneity of the leukemic SPC ROS profile in AML patient samples to determine whether more mature immunophenotypic leukemic CMP/GMP subsets might include aberrantly low ROS (putative LSC-enriched [2]) populations.

CD34^+^ AML (cases with >5% blast cells being CD34^+^) BM samples (n = 29) consisted of either abnormally expanded LMPP-/GMP-like populations (>90% of CD34^+^ blasts, in 15/29 samples), MPP-/CMP-like populations (>90% of blasts, n = 6) (examples in S1 Fig), GMP-like (>99% of blasts, n = 1), CMP-/GMP-like (>99% of blasts, n = 2), or a mixture of all SPC-types (n = 5). AML samples showed variable DCF-measured ROS levels that fell within the range of MFI values observed with control BMs. However, in contrast to normal SPC profiles, and despite immunophenotypic heterogeneity, co-existing CD34^+^CD38^low^ and CD34^+^CD38^high^ leukemic-SPCs in AML BM samples often had similar intracellular ROS levels (examples of LMPP/GMP-like and MPP/CMP-like AML in Fig 1C, and additional examples in S1F Fig). DCF staining was significantly lower in GMP-like (median 3.83) leukemic subsets when compared to the equivalent control SPC (Fig 2A and 2B, p<0.0001).

AML blasts also circulate in peripheral blood (PB) but it is unclear whether ROS profiles of leukemic-SPC are modulated by dissociation from hypoxic BM niches. We compared DCF-measured ROS levels of CD34^+^ subsets in unpaired PB (n = 43) and BM (n = 27) presentation samples and in 8 paired BM/PB AML samples. Abnormally expanded SPC observed in AML BM samples were also present in AML PB diagnosis samples. The unpaired PB vs. BM comparison showed a similar range of DCF staining between sample types (S2 Fig). Paired samples showed very similar immunophenotypes and a trend of lower DCF staining in PB in 5 out of 8 cases (S2 Fig), suggesting that the lower ROS equilibrium of some CD34^+^ AML SPC is maintained outside the hypoxic BM microenvironment and may be reduced further.

MDS with excess blasts (RAEB) patients had abnormally expanded CD34^+^CD38^low^ cells in 13 of 14 samples tested (8/9 BM, 5/5 PB) (example in S1D Fig). Most of these samples showed low DCF staining in all CD34^+^ subsets (examples in Fig 1D), as observed in AML samples, while one sample had low DCF staining in all CD34^+^CD38^low^ subsets but retained high DCF levels in CD34^+^CD38^high^ GMP (examples in S1G Fig). In contrast, MDS without excess blasts (MDS-noEB) samples (n = 12), mostly showed higher DCF staining across CD34^+^ subsets, particularly in GMP fractions (examples in Fig 1E and S1H Fig, summarised data in Fig 2A and 2B). Thus, the transition of myeloid-committed CD34^+^CD38^high^ SPCs to lower ROS appears to occur later in pre-leukemic progression.

### ROS levels in control, AML and MDS CD34^−^ subsets

We then investigated whether the altered ROS profile of AML CD34^+^SPCs is also adapted in the immunophenotypically more mature CD34^−^ blast compartment from CD34^−^ AML samples (defined as cases where <5% blasts cells are CD34^+^) (n = 23).

First, normal BM CD34^−^ CD117^+^ cells (more differentiated hematopoietic precursors) were analysed, separated into CD45RA^+^ and CD45RA^−^cells to enrich for myeloid and erythroid precursors respectively, (confirmed by CD33 and CD71/CD235a staining). CD45RA^+^ cells were DCF-high (median DCF MFI 6.54) while CD45RA^−^cells had intermediate DCF staining (MFI 4.37) (Fig 2C). In comparison only 3 of 23 CD34^−^ AMLs had CD34^−^ CD117^+^ DCF-high progenitors (>MFI 5.0), and overall the CD34^−^ CD117^+^ cells were DCF-low when compared with their normal immunophenotypic counterparts whether CD45RA^+^ or CD45RA^−^(Fig 2C) with no difference observed between unpaired BM (n = 10) and PB (n = 11) samples (S2 Fig).

Similarly to AML, CD34^−^ CD45RA^+^ as well as CD34^−^ CD45RA^−^cells were DCF-low in MDS-RAEB samples. Interestingly, in MDS—noEB DCF staining of CD34^−^CD45RA^−^cells (erythroid-enriched precursors) was significantly higher (p = 0.03) than normal CD34^−^ CD45RA^−^cells (Fig 2C).

### ROS levels in AML genetic subgroups

ROS have previously been linked to leukemogenesis of core binding factor (CBF)-AML [31] and genomic instability of *Flt3*ITD^+^ AML [15,32]. We therefore assessed the relative DCF-measured ROS levels in these and *NPM1* genetic subgroups (cytogenetic/molecular patient characteristics in S1 Table) in the dominant blast population (CD34^+^ in CD34^+^AML; n = 70 [29 BM/ 41 PB], or CD34^−^ CD117^+^ in CD34^−^ AML; n = 23 [10 BM/ 13 PB]).

**Fig 4:**
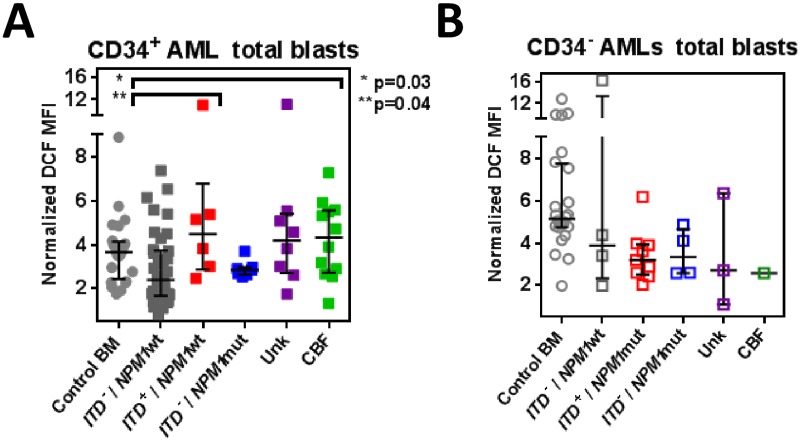
ROS levels in blast cells of different AML sub-groups. DCF levels in AML blasts (gated by CD45/SSC/CD117/CD34). Data is shown for total CD34^+^ blasts in CD34^+^AMLs (n = 70) (A) and total CD34^−^ CD117^+^ blasts in CD34^−^ AMLs (n = 23) (B) compared to the equivalent blast subset of control BMs (n = 24). AML samples, except for CBF-AMLs (CD34^+^, n = 12; CD34^−^, n = 1), were grouped according to *Flt3*ITD^+^*/NPM1* mutational status: ITD^−^*/NPM1wt*, (CD34^+^, n = 37; CD34^−^, n = 4), ITD^+^*/NPM1*wt (all CD34^+^, n = 6), ITD^-–^*/NPM1*mut (CD34^+^, n = 7; CD34^−^, n = 4), ITD^+^*/NPM1*mut, (all CD34^−^, n = 9). Unk = represents patient samples lacking mutational data.

In CD34^+^ AMLs (Fig 4A), CBF-AMLs (n = 4) had globally higher ROS levels than *Flt3*ITD^−^/*NPM1*wild type (wt) patients (MFI 4.61 vs 2.28, p = 0.03). *Flt3*ITD^+^*/NPM1*wt cases (n = 6) overall also displayed significantly higher ROS than *Flt3*ITD^*–*^*/NPM1*wt AMLs (n = 44) (MFI 4.48 vs 2.28, p = 0.04) in total CD34^+^ cells and a trend to higher than *Flt3*ITD^*–*^*/NPM1*mutated CD34^+^AMLs (n = 7) (MFI 4.48 vs 2.86; p = 0.07). In 3/6 of *Flt3*ITD^+^*/NPM1*wt patients, all SPC types were ROS-high, with DCF MFI of >5.0, the 75% percentile for all patients (S2 Fig).

In this cohort all *Flt3*ITD^+^*/NPM1*mutated patients (n = 9) were CD34^−^ AMLs (Fig 4B). These had overall lower ROS levels in their blasts (MFI 3.19) comparable to *Flt3*ITD^−^*/NPM1*mutated CD34^−^ AML blasts **(**MFI 3.34). These results suggest that a co-existing *NPM1*mutation may mitigate the increased ROS levels associated with *Flt3*ITD^+^AML.

### Combined analyses of BCL2 / ki67 with ROS in control and AML/MDS SPCs

**Fig 5:**
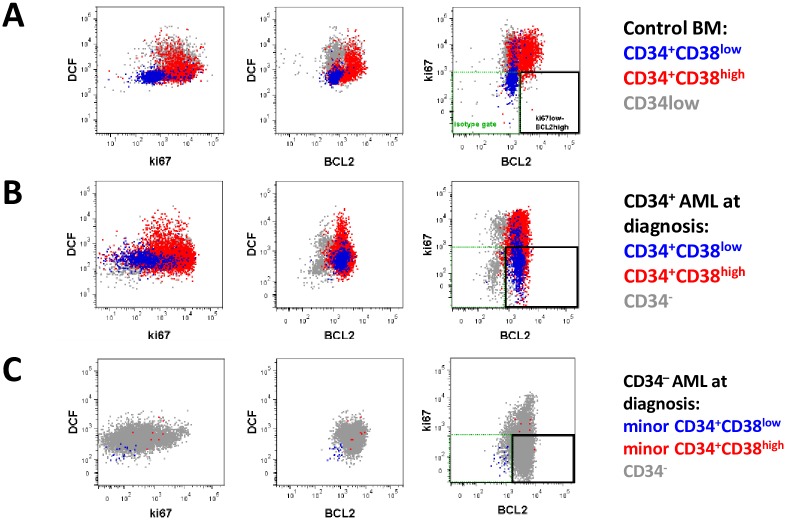
Combined ROS, ki67 and BCL2 staining of control and AML stem/progenitor cell subsets. Plots show representative immunophenotypic blasts stained with DCF, ki67 and BCL2 in control BM (A) CD34^+^AML BM (B) and CD34^−^ AML BM (C). Isotype controls for each sample were performed to establish negative gates (shown as green dotted rectangle). An expanded population with pro-survival phenotype, ki67^low^BCL2^high^, is observed in both AML types (B and C) and highlighted by black rectangle.

**Fig 6:**
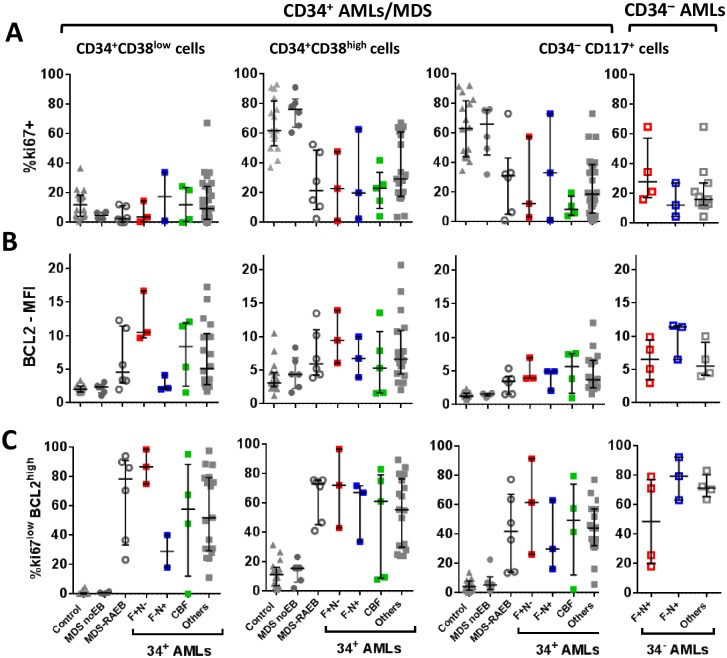
Summarised ki67 and BCL2 expression in control, AML and MDS subsets. CD34^+^CD38^low^, CD34^+^CD38^high^ and CD34^−^ (CD117^+^) blast subsets were compared for ki67-positivity (A), BCL2 expression (using BCL2-specific MFI defined by fold increase over staining with appropriate fluorescent isotype-control mAb) and the aberrant ki67^low^BCL2^high^ phenotype (C), which is defined using isotype control staining (see S3E Fig). Data includes 16 control BM, 29 CD34^+^ diagnosis AML samples (mixed BM/PB), 6 MDS with no excess blasts (no EB) and 6 MDS-RAEB samples. The CD34^−^ plots include data from 11 CD34^−^ diagnosis AMLs. Median expression and interquartile range is shown on each plot. Data is shown for all AML patients (grey squares, filled for CD34^+^ and open for CD34^−^ AMLs) and genetic subgroups. F+N- denotes ITD^+^/*NPM1*wt patients (red filled squares, all CD34^+^), F+N+ denotes ITD^+^/*NPM1*mut patients (red open squares, all CD34^−^) and F-N+ denotes ITD^−^*/NPM1*mut (blue filled open/blue open squares for CD34^+^/CD34^−^ respectively). CBF-AMLs are shown as green squares.

Although ROS, BCL2 expression and cell-cycle status have previously been functionally linked [2,23,33,34] there are as yet no data assessing these therapeutically relevant parameters in parallel for normal or leukemic SPC subsets. We therefore developed and performed a combined assay on control BM (n = 16), AML diagnosis samples (n = 40) and MDS samples (n = 12). In control BMs CD34^+^CD38^high^ cells expressed higher levels of both ki67 and BCL2 than CD34^+^CD38^low^ cells (example in Fig 5A). Closer inspection (in 11 cases) showed that although there was no significant difference in ki67 between CMP, GMP and MEP subsets, BCL2 expression followed a GMP>CMP>MEP hierarchy (S3A and S3B Fig) as observed for ROS levels. Within the more immature CD34^+^CD38^low^ fraction, all the subsets had low BCL2 but LMPP had high ki67 expression unlike the quiescent HSC/MPP populations (S3A and S3B Fig). CD34^−^ cells (mixed myeloid/erythroid) in controls were as proliferative as CD34^+^CD38^high^ SPCs but had low BCL2 at levels similar to CD34^+^CD38^low^ SPCs and MEP (Fig 6A and 6B). The observed BCL2 upregulation in the GMP subset suggests that at this myeloid maturation stage when higher ROS levels prime for proliferation/maturation, BCL2 might be transiently expressed for protection against further oxidative stress.

In CD34^+^ AML diagnosis samples (n = 29), a range of ki67 and BCL2 staining was observed across CD34^+^CD38^low^, CD34^+^CD38^high^ and CD34^−^ blast subsets. Overall, however, the blasts were less proliferative (ki67^low^) and, in some cases, over-expressed BCL2 compared to respective control subsets (Fig 6A–6C, S3C and S3D Fig). BCL2 expression was particularly high (compared to control SPC) in the LMPP-like (CD34^+^CD38^low^) populations of several LMPP/GMP-like AMLs as well as ki67^low^ including all the *Flt3*ITD^+^/*NPM1*wt patients (S3C and S3D Fig). CD34^−^AMLs (n = 11) were also significantly less proliferative and higher for BCL2 than control CD34^−^ CD117^+^ cells (Fig 5C).

Although MDS-noEB (n = 6) had a normal profile, MDS-RAEB CD34^+^ SPCs (n = 6) were similar to AMLs with a major aberrant ki67^low^BCL2^high^ population in both CD34^+^CD38^low^ and CD34^+^CD38^high^ compartments in all samples, and for some patients (4/6) also in the CD34^−^ compartment (Fig 6C). We therefore identified ki67^low^BCL2^high^ phenotype as an aberrant blast profile in both CD34^+^ and CD34^−^ AMLs and MDS-RAEB but not at the pre-leukemic stage of MDS-noEB.

There was heterogeneity in the ki67^low^BCL2^high^ population frequency between AML samples, both for total blasts and for the immunophenotypic blast subsets (S4A and S4B Fig). The high frequency in the *Flt3*ITD^+^/*NPM1*wt immature CD34^+^CD38^low^ (LMPP) SPCs was consistent with ki67^low^BCL2^high^ as a putative treatment-resistant ‘LSC-type’ subpopulation. The *Flt3*ITD^−^*/NPM1*mutated CD34^−^ AMLs, however, also all had a high ki67^low^BCL2^high^ blast frequency (Fig 6C). Since the *Flt3*ITD^−^*/NPM1*mutated subgroup is associated with treatment sensitivity it is likely that other functional properties attenuate any relative chemo-resistance from the ki67^low^BCL2^high^ phenotype, at least for this more mature CD34^−^ AML.

**Fig 7:**
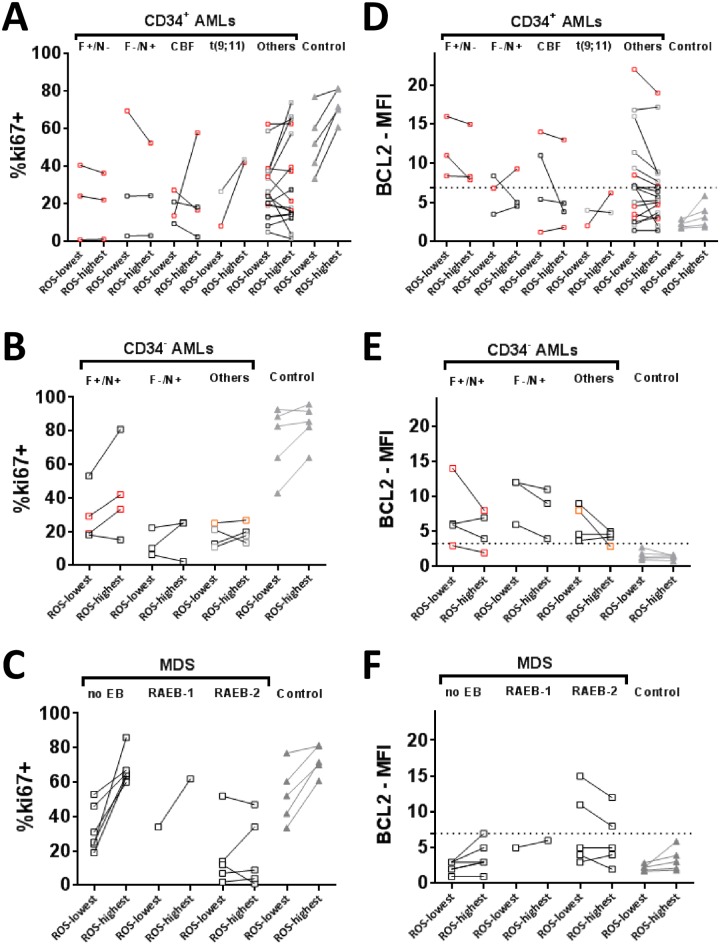
ROS-separated ki67 and BCL2 expression in AML stem/progenitor cells. AML progenitors stained with mAb for surface markers, DCF, ki67 and BCL2 were separated into ROS^lowest^ and ROS^highest^ cells based on the 20% dimmest and 20% brightest DCF staining populations respectively. Charts show ki67 and BCL2 expression respectively in ROS^lowest^ and ROS^highest^ subsets in (A-B) CD34^+^ AMLs, (C-D) in CD34^−^ AMLs and (E-F) in CD34^+^ cells in MDS patients with no excess blasts (no EB) and MDS patients with excess blasts (RAEB-1/RAEB-2). Profiles from 5 representative control/normal BMs are shown for CD34^+^ and CD34^−^CD117^+^ cells in the respective charts (grey triangles). AML patients were subdivided into *Flt3*ITD^+^/*NPM1*wt (simplified to F+/N-), *Flt3*ITD^−^/*NPM1*mut (F-/N+), *Flt3*ITD^+^/*NPM1*mut (F+N+), CBF-AMLs, t(9;11)-AMLs and other cases. AML patients with poor early response (refractory/ early-relapse/ delayed remission) indicated by red squares. Patients achieving stable remission after one course of treatment are shown as black squares. Patients for whom early outcome data was unavailable are shown as grey squares. Thresholds for high BCL2 expression are shown as dashed lines on each BCL2 chart. The threshold was set at >mean+2SD of normal CD34^+^ (>6.9) or CD34^−^ blasts (>3.3).

We hypothesised that BCL2 over-expression (with downstream effects on apoptosis, metabolic regulation and cell-cycle) might be maintained in ROS-high blasts of some AMLs to counteract further oxidative stress similar to the observed BCL2 upregulation at the GM maturation stage. We gated on blasts with 20% highest DCF fluorescence and 20% lowest DCF fluorescence to allow a comparison between ROS^highest^ and ROS^lowest^ blasts (AML n = 40, MDS n = 12). There was a relative enrichment of the more immature immunophenotypic SPC (hierarchy LMPP/MPP >CMP/GMP) in the ROS^lowest^ vs ROS^highest^ fractions for most of the CD34^+^AMLs (n = 24) (S5A Fig) but no SPC correlation with a specific ki67/BCL2 profile or overall high ROS levels. Interestingly ROS^lowest^/ROS^highest^ gating of CD34^+^ SPCs in MDS-noEB revealed an aberrant relative expansion of CD34^+^CD38^low^ and MEP/CMP SPCs in the ROS^lowest^ and ROS^highest^ fractions respectively (S5B Fig). Although some patients had lower ki67 in ROS^lowest^ fractions as observed for normal BM and MDS-noEB (Fig 7A–7C), the majority did not have ROS^low^ enrichment of the LSC-like ki67^low^BCL2^high^ phenotype (S6 Fig). Similar ki67 and BCL2 levels were, however, maintained between ROS^lowest^ and ROS^highest^ blasts in a number of AML/MDS-RAEB patients. From available clinical data of early post-treatment outcome for CD34^+^ AMLs, high BCL2 over-expression (>6.9-fold above isotype) in ROS^highest^ as well as ROS^lowest^ fractions seemed to be most associated with initial treatment resistance (Fig 7D) although numbers were limited. A correlation between BCL2 overexpression in ROS^lowest^/ROS^highest^ and early treatment response was not apparent in CD34^−^ AMLs including the *Flt3*ITD^+^/*NPM1*mutated patients (Fig 7E).

BCL2 inhibition experiments were performed using ABT199 in a subgroup of pre-treatment AMLs (n = 9). High levels of ABT199 sensitivity (EC50 at or below 100nM) was observed in both CD34^+^ and CD34^−^ AMLs, irrespective of differences in intracellular ROS, BCL2 and cell-cycle status. ABT199-induced cell death was greater than that observed using ara-C for 5 of 6 samples (4/4 CD34^+^ AMLs and 1/2 CD34^−^ AMLs) where the drug assays were performed in parallel (S7A and S7B Fig). In the 2 samples tested, the potentially LSC-enriched, immature CD34^+^CD38^low^ blast fractions showed equivalent or greater ABT199 sensitivity than more mature CD34^+^CD38^high^ blasts (S7C Fig) and had a similar profile of differential sensitivity to ABT199 compared to ara-C (S7D Fig).

## Discussion

This study provides a phenotypic profile for normal and leukemic SPC-subsets of functional cell properties that may, by dysregulation downstream of genetic abnormalities, promote survival and propagation in AML. Our results show decoupling of the relationship between normal myeloid maturation and cellular ROS/ BCL-2/ cell-cycle profiles in AML and MDS-RAEB with certain leukemic blast subpopulations displaying a chimeric functional phenotype of both ‘stem-cell-like’ and ‘GM-like’ properties. Normal human CD34^+^ and CD34^−^CD117^+^ SPC subsets exhibit distinct patterns of ROS, cell-cycle activity with BCL2 expression upregulation at the GM stage. These ROS/ cell-cycle patterns are lost in AML and MDS-RAEB with an aberrant ki67^low^BCL2^high^ phenotype observed in both CD34^+^ and CD34^−^ blast subpopulations at disease presentation.

By combining immunophenotypic characterisation of normal human SPC subsets with a ROS assay we show that although the DCF-low/ROS-low state has been associated with greater quiescence and the DCF-high/ROS-high state with proliferation and differentiation in primitive hematopoietic cells [20], the more mature (CD34^+^CD38^high^) progenitors actually contain a combination of ROS-low and ROS-high cells with MEP consistently ROS-low, and GMP being ROS-high while CMP are a mixture of ROS-high^/^low cells. Colony assay data support a model whereby ROS-high CMP are pre-committed to become GMP while ROS-low CMP are either less committed and/or pre-committed to MEP. This ROS-based separation of human CMP is consistent with ROS being a driver of myeloid differentiation [6,7,35]. Extending our analysis by evaluation of the combined ROS/cell-cycle/BCL2 profile showed that all subsets (GMP/CMP/MEP) of CD34^+^CD38^high^ cells had high levels of cell-cycle activity despite variation in ROS levels but BCL2 upregulation correlated with increasing ROS levels and was thus myeloid-specific. BCL2 protein upregulation may be a survival cue to counteract oxidative stress-induced apoptosis in GMPs when higher ROS levels are required to prime for proliferation/maturation. It could also be speculated, however, to predispose GMP to accumulating more DNA damage [36] and explain the relative expansion of GMP rather than CMP as observed in most MDS-RAEB and AML patients [28,37].

All normal CD34^+^CD38^low^ SPCs were BCL2^low^ and, as expected, HSC and MPP were ROS-low ki67^low^ (quiescent) but interestingly the infrequent LMPP population appeared less ‘stem-cell-like’ with higher ROS and greater cell-cycle activity. The immunophenotypically immature SPCs of AML and MDS-RAEB samples had the lowest ROS levels but the overall ROS profile was less heterogeneous compared to normal; the CD34^+^CD38^high^ (GMP-like in particular) and CD34^−^AML ‘mature’ blasts were both lower in ROS than the immunophenotypic equivalent control SPCs. This shift towards immature SPC ROS levels may contribute to the differentiation block in AML, consistent with data showing that agents increasing ROS can induce maturation of AML blasts [38,39]. The ROS-low pattern was observed, often more distinctly, in AML cells in PB and may represent an adaptation of AML sub-clones in BM that traffic to the PB [40] akin to that of HSPC preserving their hypoxic status while circulating in the normoxic blood environment [41].

While intracellular ROS levels varied in our cohort (~12-fold range), this was lower than the variation reported for extracellular ROS produced by AML blasts (4-log range) using different methods of ROS detection [18]. It is plausible that extracellular ROS production is also high in our patients while intracellular ROS is kept under control by an enhanced anti-oxidant response [42,43], which may be targetable for inhibition [44–45]. Our data partially support the link between *Flt3*ITD^+^ mutation and higher ROS levels [15] but also show that ROS levels were lower in most *NPM1-*mutated/ *Flt3*ITD^+^ cases (all CD34^−^ AML in our cohort). Though patient numbers are low, this could suggest that the *NPM1*-mutated phenotype mitigates the effect of *Flt3*ITD^+^ on intracellular ROS levels, resulting in a lower risk of DNA damage and progression as suggested by clinical data [46]. Higher ROS levels were also observed in some CBF-AMLs which is interesting as ROS signalling is crucial for maintaining AML1-ETO fusion protein-induced aberrant progenitor cells in a drosophila model [31]. The elevated ROS of CBF-AMLs may be related to both granulocytic maturation and enhanced sensitivity to chemotherapy by the mechanism of ROS-induced apoptosis.

We also detected higher ROS than controls in SPCs of MDS-noEB, most marked in the erythroid precursor-enriched subset (CD117^+^CD34^−^CD45RA^−^). This may be linked with the reported higher apoptosis and cytopenias observed in MDS patients [37] from increased oxidative stress particularly of the erythroid lineage. Reduced ki67 expression, high BCL2 expression and most significantly an expanded ki67^low^BCL2^high^ phenotype were observed in AML and MDS-RAEB but not MDS-noEB. While the ki67^low^BCL2^high^ aberrant phenotype was highly expanded in *Flt3*ITD^+^ cases including in the LSC-enriched LMMP-like subpopulation [28], it was also present in other AML subtypes. The in vivo LSC frequency of identified ki67^low^BCL2^high^cells could not be investigated by xenografting experiments due non-viability following intracellular staining but ki67 and BCL2 expression, either as single parameters or when combined as ki67^low^BCL2^high^, did not correlate with early outcome to standard treatment in our small patient group. This is not unexpected as chemo-resistance is also dependent on the balance between activities of different pro-/anti-apoptotic BCL2 family proteins [47–49] as well as drug efflux activity [50]. Further sub-analysis of ROS^highest^ and ROS^lowest^ cells within the blast population of each patient showed that, although aberrant BCL2 overexpression has been reported as an adaptation of LSC-enriched ROS^lowest^ AML cells [2], ROS^highest^ cells were also BCL2^high^ for some CD34^+^ AMLs (8/24) and CD34^−^ AMLs (7/9). Intriguingly, aberrant overexpression of BCL2 in ROS^highest^ as well as ROS^lowest^ blasts was the profile most associated with early chemotherapy resistance from limited available data in the 24 CD34^+^ AMLs with outcome data. However, sensitivity to BCL2 inhibition *in vitro* using ABT199 in a small number of tested patients was high across both CD34^+^ and CD34^-^ AML blast types with different ROS, ki67 and BCL2 levels. Furthermore AML blasts including potentially LSC-enriched fractions, showed greater sensitivity to ABT199 than ara-C. Thus BCL2 dependence was shared by phenotypically different AML blasts. This in vitro data is supportive of greater clinical efficacy of BCL2-inhibition therapy across different AML subtypes compared to standard chemotherapy.

It may also be postulated from our results that BCL2 over-expression (with downstream effects on apoptosis, metabolic regulation and cell-cycle) is an adapted ‘GM-like’ property (to intrinsic or extrinsic dysregulation) that in some AMLs modulates the apoptotic threshold to additional oxidative stress as well as metabolic adjustments. This could contribute to relative chemo-resistance by protecting leukemic blasts with higher ROS from apoptosis triggered by chemotherapy-induced oxidative damage.

## Supporting Information

S1 Fig Immunophenotypic characterisation of stem/progenitor cell populations. (PDF)

S2 Fig Comparing ROS levels between AML marrow and blood diagnosis specimens. (PDF)

S3 Fig ki67 and BCL2 expression in immunophenotypic subsets. (PDF)

S4 Fig Heat maps of ki67, BCL2 and ROS levels in AML and control progenitors. (PDF)

S5 Fig Stem/progenitor cell (SPC) composition of ROS-separated AML, MDS and control blasts. (PDF)

S6 Fig Analysis of ROS^low^ and ROS^high^ progenitors in AML. (PDF)

S7 Fig BCL2 inhibition induces toxicity in AML blasts from CD34^+^ and CD34^−^ AMLs. (PDF)

S1 File Supplementary Information & Methods. (DOCX)

S1 Table AML patient /sample characteristics. (DOCX)

S2 Table Monoclonal antibody and fluorochrome panels used in combined assays. (DOCX)
