# Supplementary material for: Normal Hematopoietic Progenitor Subsets Have Distinct Reactive Oxygen Species, BCL2 and Cell-Cycle Profiles That Are Decoupled from Maturation in Acute Myeloid Leukemia
Source: PLoS One. 2016 Sep 26;11(9):e0163291. doi: 10.1371/journal.pone.0163291 (PMC5036879; doi:10.1371/journal.pone.0163291)
Supplement: S1 Fig — (PDF) [file pone.0163291.s001.pdf]

S1 Figure

Immunophenotypic characterisation of stem/progenitor cell populations

Flow cytometry plots showing gating of stem/progenitor cell (SPC) subpopulations (left hand column, following gating of $CD117^{+}SSC^{low}$ cells to exclude lymphoid and more mature myeloid cells). CD34 vs. CD38 staining is analysed to derive immature $CD34^{+}CD38^{low}$ (highlighted by red boxes) and less immature $CD34{+}CD38^{high}$ cell populations (highlighted by blue boxes). These were then analysed further to derive HSC, MPP and LMPP populations (from $CD34^{+}CD38^{low}$ cells; middle column) and CMP, GMP and MEP populations (from $CD34^{+}CD38^{high}$ cells; right hand column). Examples of control BM (A), pre-treatment adult AML (B-C), MDS-RAEB (D) and MDS-no excess blasts (noEB) (E) are shown.

Histograms of DCF staining of different immunophenotypic SPCs overlaid with lymphocytes within the sample show differential ROS levels between SPC types. Example AMLs (F) and MDS-RAEB (G), and MDS-noEB samples (H) are shown with normalised ROS values indicated for each subset. These were calculated after DCF MFI values for each SPC type were divided by DCF MFI of lymphocytes within the same sample. The SPC type is indicated by colouring scheme of labels. Granulocyte/macrophage progenitor (GMP) = green, Common myeloid progenitor (CMP) = orange, megakaryocyte-erythroid progenitor (MEP) = red dashed, lymphoid-primed multi-potent progenitor (LMPP) = blue, multi-potent progenitor (MPP) = grey dashed, haematopoietic stem cell (HSC) = black.

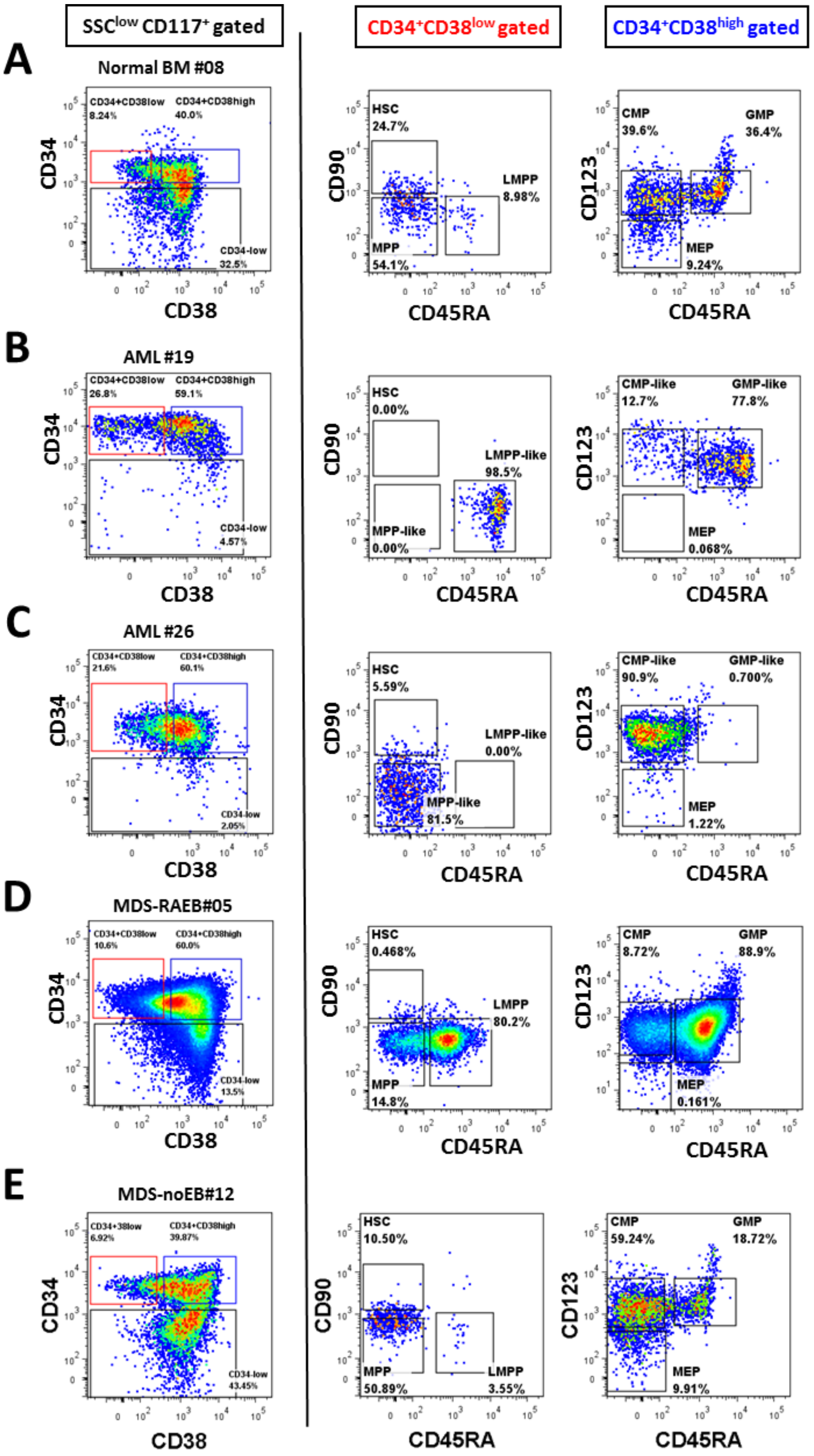
SSClow CD117+ gated
CD34+CD38low gated
CD34+CD38high gated
A
Normal BM #08
CD34+CD38low 8.24%
CD34+CD38high 40.0%
CD34-low 32.5%
CD34
CD38
HSC 24.7%
LMPP 8.98%
MPP 54.1%
CD90
CD45RA
CMP 39.6%
GMP 36.4%
MEP 9.24%
CD123
CD45RA
B
AML #19
CD34+CD38low 26.8%
CD34+CD38high 59.1%
CD34-low 4.57%
HSC 0.00%
LMPP-like 98.5%
MPP-like 0.00%
CMP-like 12.7%
GMP-like 77.8%
MEP 0.068%
C
AML #26
CD34+CD38low 21.6%
CD34+CD38high 60.1%
CD34-low 2.05%
HSC 5.59%
LMPP-like 0.00%
MPP-like 81.5%
CMP-like 90.9%
GMP-like 0.700%
MEP 1.22%
D
MDS-RAEB#05
CD34+CD38low 10.6%
CD34+CD38high 60.0%
CD34-low 13.5%
HSC 0.468%
LMPP 80.2%
MPP 14.8%
CMP 8.72%
GMP 88.9%
MEP 0.161%
E
MDS-noEB#12
CD34+38low 6.92%
CD34+CD38high 39.87%
CD34-low 43.45%
HSC 10.50%
MPP 50.89%
LMPP 3.55%
CMP 59.24%
GMP 18.72%
MEP 9.91%

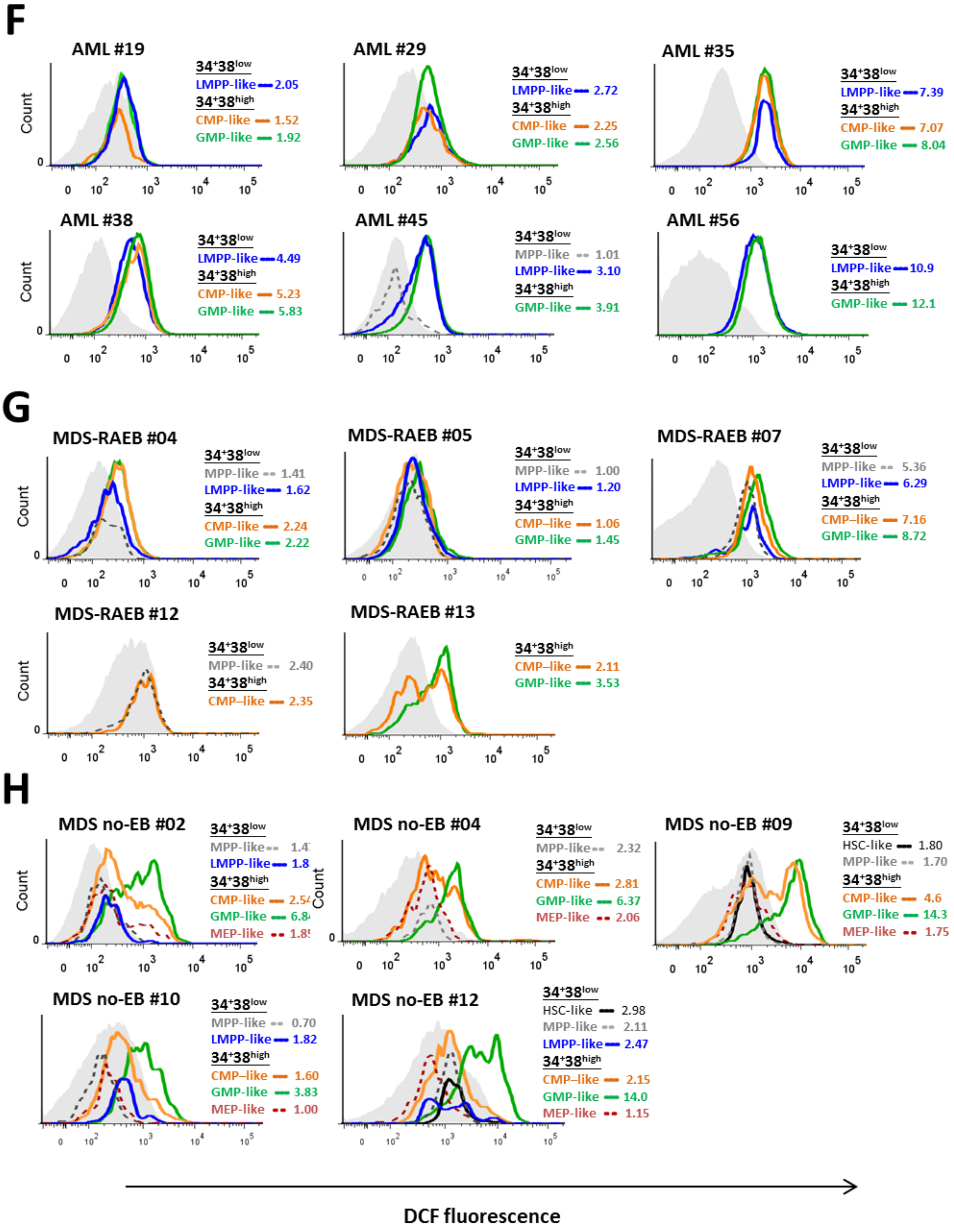
F
AML #19
34+38low
LMPP-like 2.05
34+38high
CMP-like 1.52
GMP-like 1.92
AML #29
34+38low
LMPP-like 2.72
34+38high
CMP-like 2.25
GMP-like 2.56
AML #35
34+38low
LMPP-like 7.39
34+38high
CMP-like 7.07
GMP-like 8.04
AML #38
34+38low
LMPP-like 4.49
34+38high
CMP-like 5.23
GMP-like 5.83
AML #45
34+38low
MPP-like 1.01
LMPP-like 3.10
34+38high
GMP-like 3.91
AML #56
34+38low
LMPP-like 10.9
34+38high
GMP-like 12.1
G
MDS-RAEB #04
34+38low
MPP-like 1.41
LMPP-like 1.62
34+38high
CMP-like 2.24
GMP-like 2.22
MDS-RAEB #05
34+38low
MPP-like 1.00
LMPP-like 1.20
34+38high
CMP–like 1.06
GMP-like 1.45
MDS-RAEB #07
34+38low
MPP-like 5.36
LMPP-like 6.29
34+38high
CMP–like 7.16
GMP-like 8.72
MDS-RAEB #12
34+38low
MPP-like 2.40
34+38high
CMP–like 2.35
MDS-RAEB #13
34+38high
CMP–like 2.11
GMP-like 3.53
H
MDS no-EB #02
34+38low
MPP-like 1.4
LMPP-like 1.8
34+38high
CMP-like 2.5
GMP-like 6.8
MEP-like 1.8
MDS no-EB #04
34+38low
MPP-like 2.32
34+38high
CMP-like 2.81
GMP-like 6.37
MEP-like 2.06
MDS no-EB #09
34+38low
HSC-like 1.80
MPP-like 1.70
34+38high
CMP-like 4.6
GMP-like 14.3
MEP-like 1.75
MDS no-EB #10
34+38low
MPP-like 0.70
LMPP-like 1.82
34+38high
CMP–like 1.60
GMP-like 3.83
MEP-like 1.00
MDS no-EB #12
34+38low
HSC-like 2.98
MPP-like 2.11
LMPP-like 2.47
34+38high
CMP–like 2.15
GMP-like 14.0
MEP-like 1.15
Count
DCF fluorescence
