# Supplementary material for: Normal Hematopoietic Progenitor Subsets Have Distinct Reactive Oxygen Species, BCL2 and Cell-Cycle Profiles That Are Decoupled from Maturation in Acute Myeloid Leukemia
Source: PLoS One. 2016 Sep 26;11(9):e0163291. doi: 10.1371/journal.pone.0163291 (PMC5036879; doi:10.1371/journal.pone.0163291)

S2 Figure

Comparing ROS levels between AML marrow and blood diagnosis specimens

Charts illustrating DCF staining of expanded AML stem/progenitor cells (SPC) in 29 BM and 41 PB unpaired specimens separated into $CD34^{+}CD38^{low}$ subsets (A) and $CD34^{+}CD38^{high}$ subsets (B). *Flt3ITD*$^{+}$ cases are shown as red squares and CBF-AMLs as green squares. The remaining patients are shown as grey squares for PB and black squares for BM. Representative example of immunophenotyping and normalised ROS in paired pre-treatment AML BM and PB samples (C), with light grey filled histogram indicating the reference lymphocyte population (used to generate the normalised DCF values shown for SPC). ROS levels were also compared between 8 paired marrow (black) and blood (grey) specimens, in whom dominant progenitor cells were LMPP-like/GMP-like/MPP-like and/or CMP-like (D), with patient numbers indicated under the x axis. ROS levels were also compared between $CD34^{-}$blasts of unpaired $CD34^{-}$ AML BM and PB samples after separation into $CD34^{-}$ $CD45RA^{+}$ and $CD34^{-}$ $CD45RA^{-}$ subsets (E). *Flt3*ITD^{+} cases (also all *NPM1*mut) are shown as larger red squares, *Flt3wt (*ITD^{-}*)/NPM1*mut as blue squares and CBF-AMLs as green squares. The remaining patients are shown as open grey squares.

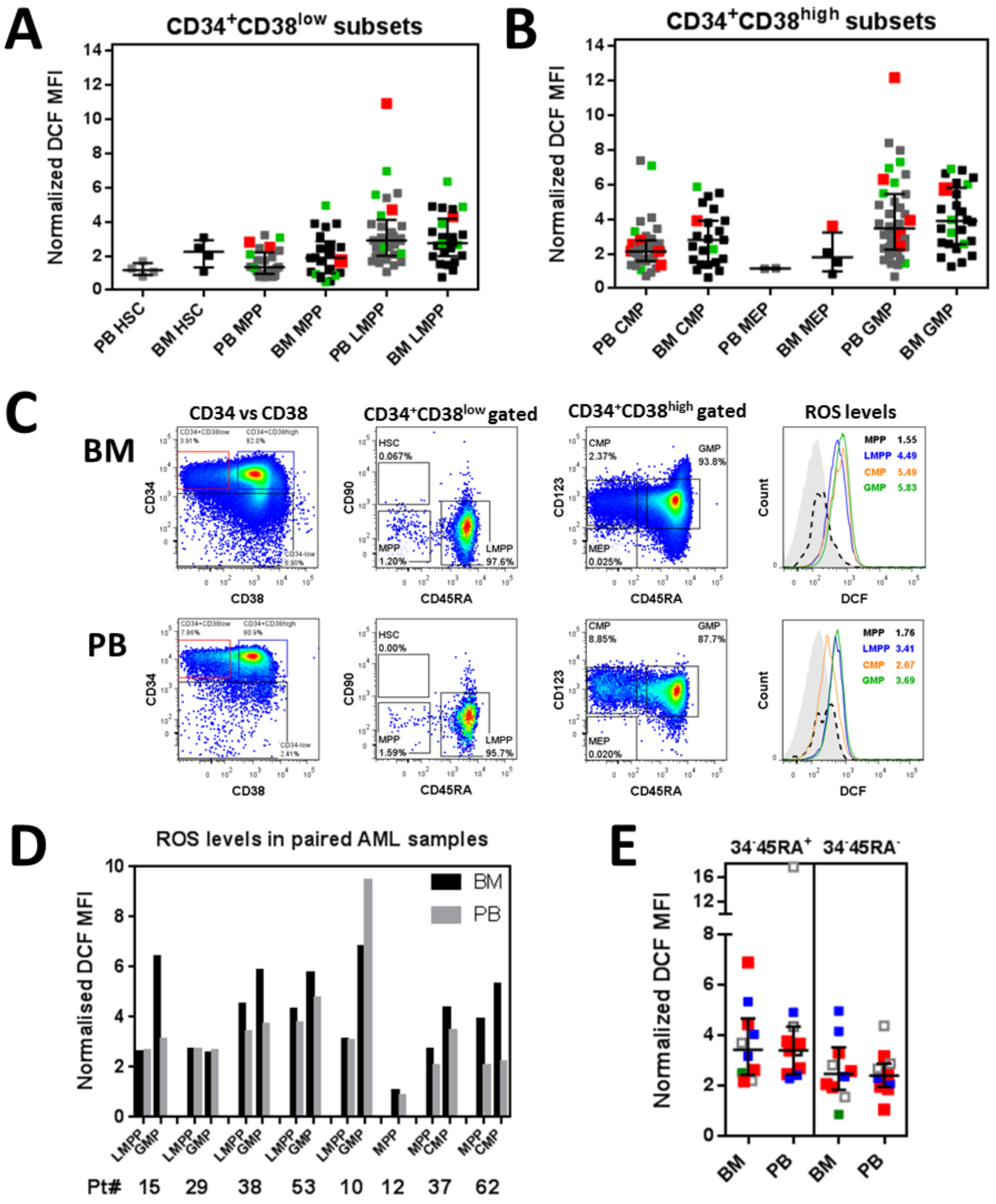

Supplement: S2 Fig — (PDF) [file pone.0163291.s002.pdf]
