# Supplementary material for: Normal Hematopoietic Progenitor Subsets Have Distinct Reactive Oxygen Species, BCL2 and Cell-Cycle Profiles That Are Decoupled from Maturation in Acute Myeloid Leukemia
Source: PLoS One. 2016 Sep 26;11(9):e0163291. doi: 10.1371/journal.pone.0163291 (PMC5036879; doi:10.1371/journal.pone.0163291)

S3 Figure

ki67 and BCL2 expression in immunophenotypic subsets

Control BM-derived HSC/MPP, CMP/MEP/GMP were compared for ki67-positivity (A) and BCL2 expression (B). AML pre-treatment/diagnosis samples with progenitors defined as MPP-like/LMPP-like and CMP-like/GMP-like assayed for ki67-positivity (C) and BCL2 expression (D). *Flt3*ITD^{+} and CBF-AMLs are shown as red and green squares respectively. Median and interquartile range is shown on each plot. ki67 versus BCL2 staining of CD34^{+}CD38^{low}, CD34^{+}CD38^{high} and CD34^{-} gated subsets of a typical pre-treatment CD34^{+} AML sample, with isotype controls used to set quadrant gates around the double negative population (E). ki67^{low}BCL2^{high} cells are calculated based on the number of cells in lower right quadrant (black box).

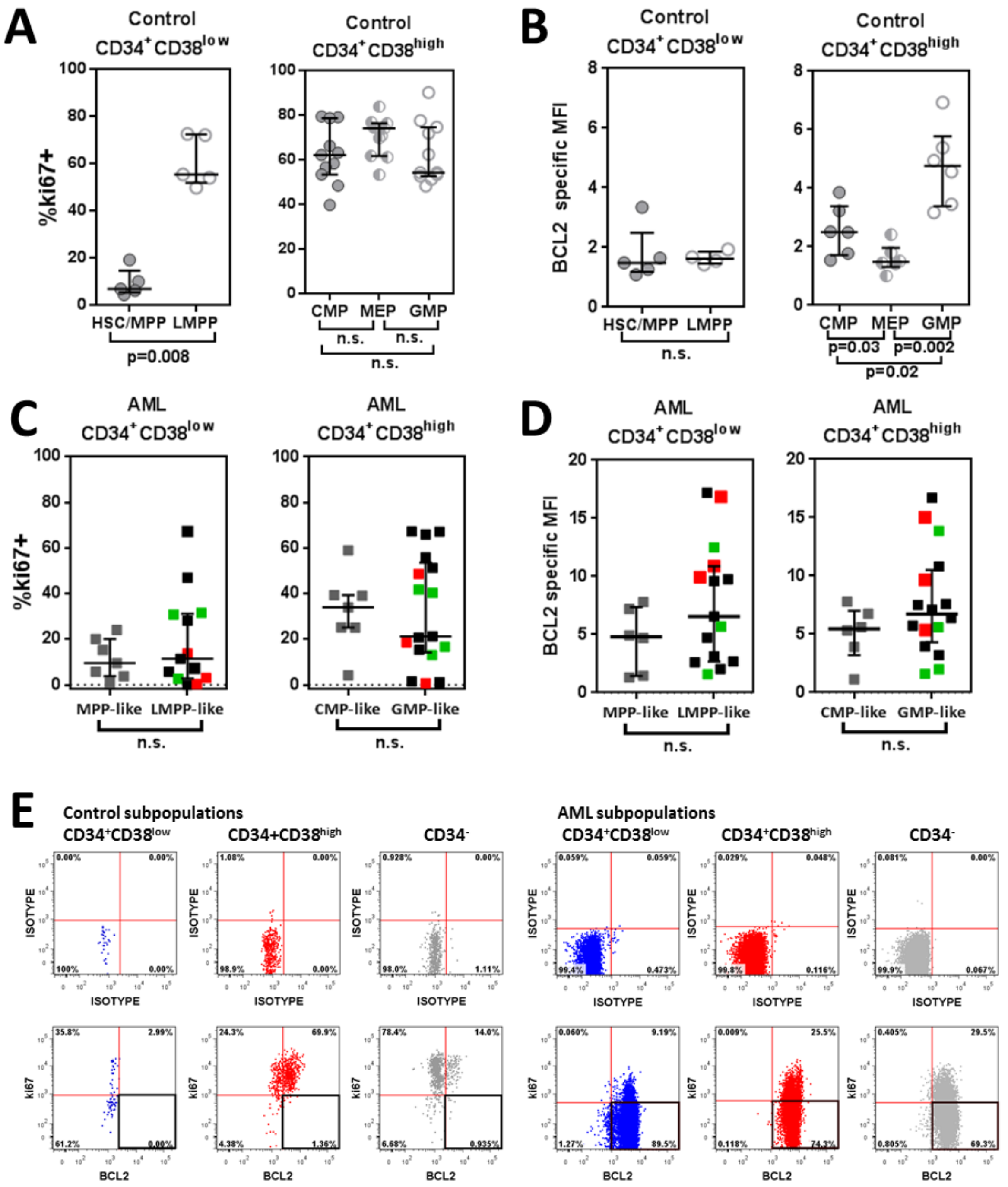

Supplement: S3 Fig — (PDF) [file pone.0163291.s003.pdf]
