# Supplementary material for: Normal Hematopoietic Progenitor Subsets Have Distinct Reactive Oxygen Species, BCL2 and Cell-Cycle Profiles That Are Decoupled from Maturation in Acute Myeloid Leukemia
Source: PLoS One. 2016 Sep 26;11(9):e0163291. doi: 10.1371/journal.pone.0163291 (PMC5036879; doi:10.1371/journal.pone.0163291)

S4 Figure

Heat maps of ki67, BCL2 and ROS levels in AML and control progenitors

Heat map showing %ki67^{low}BCL2^{high}, %ki67, BCL2 expression and ROS levels in total dominant blasts of CD34^{+} AMLs (A) and CD34^{-}AMLs (B). Control patterns are shown using CD34^{+} and CD34^{-}subsets from 5 representative normal BM samples. Colour scale for % ki67^{low}BCL2^{high} and %ki67 is dark blue (0%) to dark red (100%). Colour scale for BCL2 expression (fold increase in MFI of BCL2 staining over isotype control) is 0 (dark blue) to 10 (dark red). ROS levels represent normalised DCF MFI values of total dominant blast type, with scale at 0 (dark blue) to 7 (dark red). Patient number is indicated under each column and early outcome data is shown above each column. Outcomes are abbreviated as follows; ref=refractory, C1= morphological remission post course 1, C2= morphological remission post course 2, rel 6m= relapse within 6 months, d= death within 30 days, - = no outcome data available.

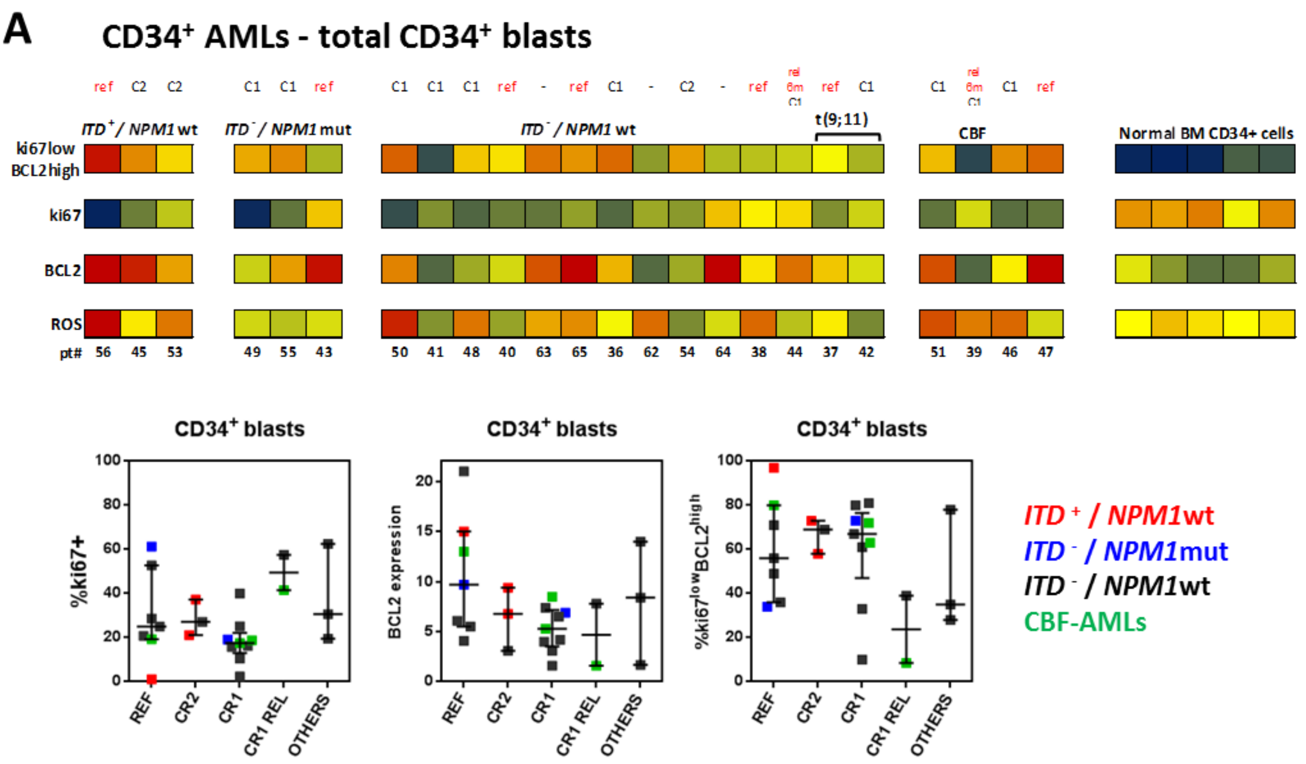

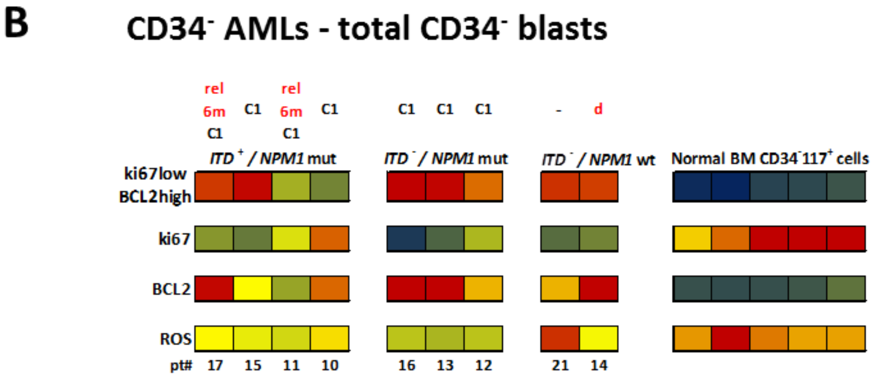

Supplement: S4 Fig — (PDF) [file pone.0163291.s004.pdf]
