# Supplementary material for: Normal Hematopoietic Progenitor Subsets Have Distinct Reactive Oxygen Species, BCL2 and Cell-Cycle Profiles That Are Decoupled from Maturation in Acute Myeloid Leukemia
Source: PLoS One. 2016 Sep 26;11(9):e0163291. doi: 10.1371/journal.pone.0163291 (PMC5036879; doi:10.1371/journal.pone.0163291)

S5 Figure

Stem/progenitor cell (SPC) composition of ROS-separated AML, MDS and control blasts. For each sample, blasts with 20% highest and 20% lowest DCF staining were gated followed by inspection for constituent progenitor populations. In $CD34^{+}$ AMLs (A), SPCs were either L=LMPP-like, M=MPP-like, C=CMP-like, G=GMP-like. Breakdown of subpopulations in MDS patients with no excess blasts (MDS no EB) (n=6), and MDS patients with excess blasts (MDS RAEB-1/RAEB-2) (n=6), and representative controls (B), HSC-like (mixed with MPP; H/M) and MEP-like populations (mixed with CMP; ME/C) were also present. Patients classified as ROS high (total blasts >5.0) have the patient ID coloured in red. Data shown for AML patients where cytogenetic and mutational information was available.

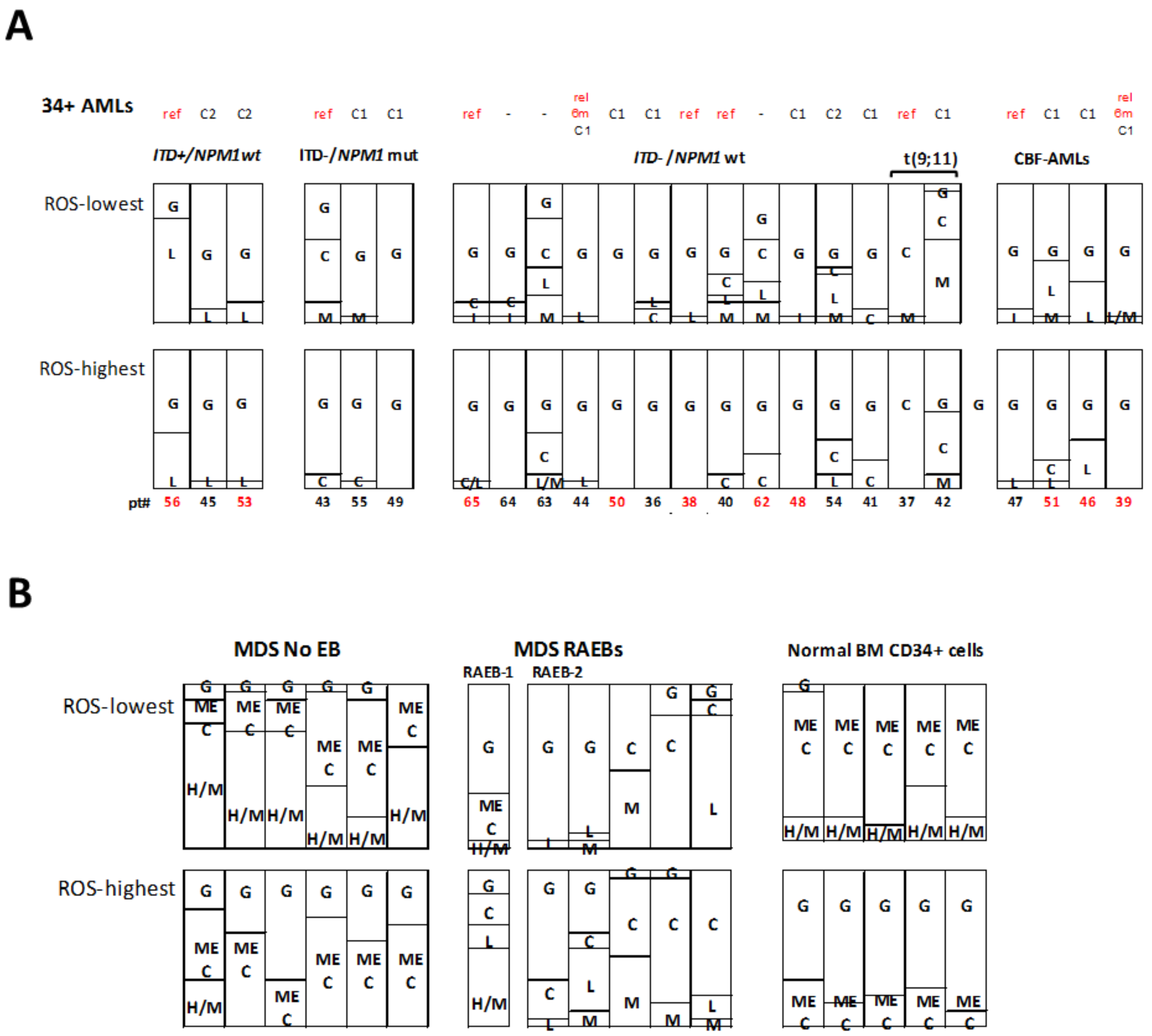

Supplement: S5 Fig — (PDF) [file pone.0163291.s005.pdf]
