# Supplementary material for: Normal Hematopoietic Progenitor Subsets Have Distinct Reactive Oxygen Species, BCL2 and Cell-Cycle Profiles That Are Decoupled from Maturation in Acute Myeloid Leukemia
Source: PLoS One. 2016 Sep 26;11(9):e0163291. doi: 10.1371/journal.pone.0163291 (PMC5036879; doi:10.1371/journal.pone.0163291)
Supplement: S6 Fig — (PDF) [file pone.0163291.s006.pdf]

S6 Figure

Analysis of ROS^{low} and ROS^{high} progenitors in AML

AML progenitors separated into ROS^{lowest} and ROS^{highest} cells (based on 20% dimmest and 20% brightest DCF staining populations respectively) are assessed for ki67^{low}BCL2^{high} frequency in (A) CD34^{+} AMLs (n=29), (B) in CD34^{-} AMLs (n=11) and (C) in CD34^{+} cells in MDS no EB (n=5) and MDS RAEB-1/RAEB-2 patients (n=6). Representative control/normal CD34^{+} and CD34^{-}CD117^{+} cells are also shown (grey triangles). AML patients were subdivided into *Flt3*ITD^{+}/*NPM1*wt (simplified to F+/N-), *Flt3*ITD^{-}/*NPM1*mut (F-/N+), *Flt3*ITD^{+}/*NPM1*mut (F+N+), CBF-AMLs, t(9;11)-AMLs and other cases. AML patients with poor early response (refractory/ early-relapse/ delayed remission) indicated by red squares. Patients achieving stable remission after one course of treatment are shown as black squares. Patients for whom early outcome data was unavailable are shown as grey squares.

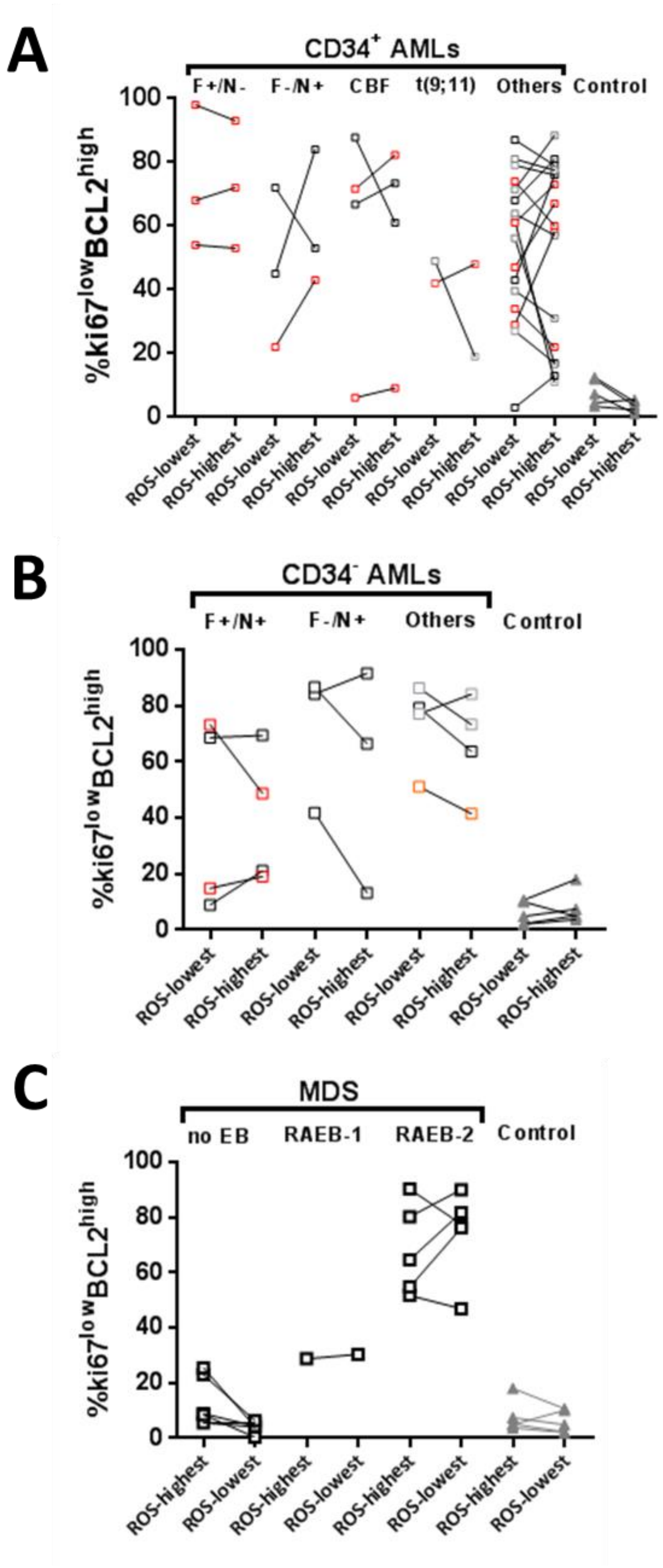
A
CD34+ AMLs
F+/N-
F-/N+
CBF
t(9;11)
Others
Control
%ki67lowBCL2high
100
80
60
40
20
0
ROS-lowest
ROS-highest
B
CD34- AMLs
F+/N+
F-/N+
Others
Control
C
MDS
no EB
RAEB-1
RAEB-2
Control
