# Supplementary material for: Normal Hematopoietic Progenitor Subsets Have Distinct Reactive Oxygen Species, BCL2 and Cell-Cycle Profiles That Are Decoupled from Maturation in Acute Myeloid Leukemia
Source: PLoS One. 2016 Sep 26;11(9):e0163291. doi: 10.1371/journal.pone.0163291 (PMC5036879; doi:10.1371/journal.pone.0163291)
Supplement: S7 Fig — (PDF) [file pone.0163291.s007.pdf]

S7 Figure

BCL2 inhibition induces toxicity in AML blasts from $CD34^{+}$ and $CD34^{-}$ AMLs

Pre-treatment AML mononuclear cells were incubated with ABT199 or ara-C at the shown concentrations for 16hrs and 48hrs respectively. Cells were stained for AML blast markers and Annexin-V and 7AAD to allow live (Annexin-V^{neg} $7AAD^{neg}$) / apoptotic and dead (Annexin-V^{pos}/$7AAD^{pos}$) cell discrimination. In vitro drug sensitivities were assessed in 4 different $CD34^{+}$ AMLs (A) and 2 different $CD34^{-}$ AMLs (B), after gating on the immunophenotypic blast population ($CD45^{int}CD117^{+}$ for $CD34^{-}$AMLs). In all examples, total blasts cells were more sensitive to ABT199 than ara-C. Dashed lines indicate the concentration of drug inducing half maximal toxicity (EC50). In each case, the immunophenotypic blast type, ROS levels, BCL2 and ki67 levels of total blasts are shown.

ABT199 sensitivity was also compared between co-existing immature $CD34^{+}CD38^{low}$ blasts and more mature $CD34^{+}CD38^{high}$ blasts of 2 different $CD34^{+}$ AMLs (C), with immunophenotypic blast type, ROS levels, BCL2 and ki67 levels of immature and mature blasts shown for cases tested.

Sensitivities to ABT199 and ara-C in immature potentially LSC-enriched $CD34^{+}CD38^{low}$ blasts were also assessed in 2 different CD34+ AMLs (D).

KEY: Immunophenotypic blast type for CD34+ AMLs is shown where LMPP/GMP= lymphoid primed multipotent progenitor / granulocyte-macrophage progenitor –like subsets are >90% of total blasts, MPP/CMP = multipotent progenitor / common myeloid progenitor –like subsets are >90% of total blasts. Mixed SPC = stem/progenitor cell mixture of LMPP/GMP and MPP/CMP where no single subset exceeds 80% of total blasts. ROSn: normalised ROS levels based on DCF MFI of blasts/DCF MFI of lymphocytes. BCL2n: normalised BCL2 expression based on BCL2 MFI /isotype control MFI. ki67+; % of blasts staining positive for intracellular ki67 after subtraction of isotype control staining.

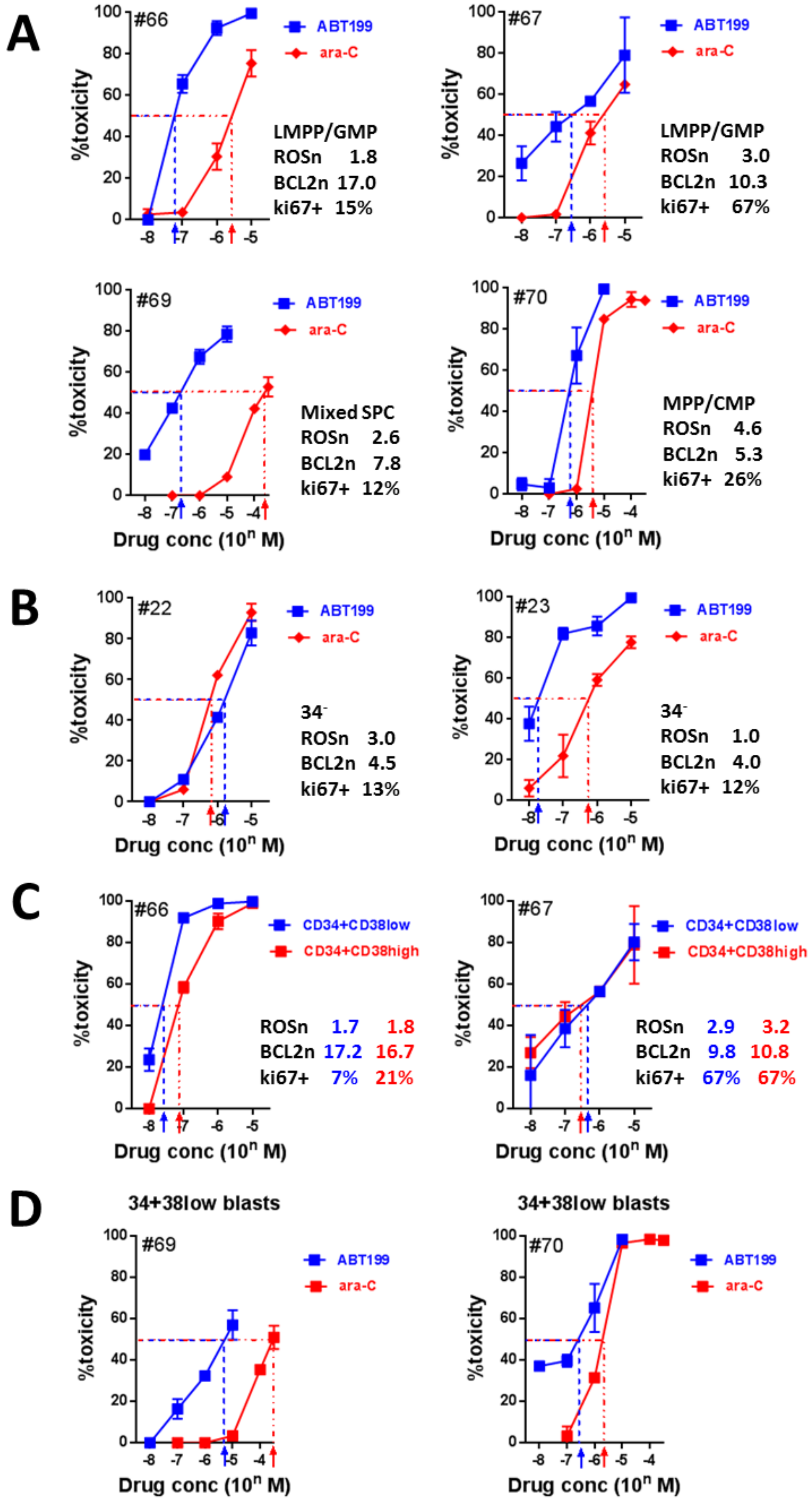
A
#66
ABT199
ara-C
%toxicity
LMPP/GMP
ROSn 1.8
BCL2n 17.0
ki67+ 15%
#67
LMPP/GMP
ROSn 3.0
BCL2n 10.3
ki67+ 67%
#69
Mixed SPC
ROSn 2.6
BCL2n 7.8
ki67+ 12%
#70
MPP/CMP
ROSn 4.6
BCL2n 5.3
ki67+ 26%
Drug conc (10^n M)
B
#22
34-
ROSn 3.0
BCL2n 4.5
ki67+ 13%
#23
34-
ROSn 1.0
BCL2n 4.0
ki67+ 12%
C
#66
CD34+CD38low
CD34+CD38high
ROSn 1.7 1.8
BCL2n 17.2 16.7
ki67+ 7% 21%
#67
ROSn 2.9 3.2
BCL2n 9.8 10.8
ki67+ 67% 67%
D
34+38low blasts
#69
#70
