# Supplementary material for: Normal Hematopoietic Progenitor Subsets Have Distinct Reactive Oxygen Species, BCL2 and Cell-Cycle Profiles That Are Decoupled from Maturation in Acute Myeloid Leukemia
Source: PLoS One. 2016 Sep 26;11(9):e0163291. doi: 10.1371/journal.pone.0163291 (PMC5036879; doi:10.1371/journal.pone.0163291)
Supplement: S1 File — (DOCX) [file pone.0163291.s008.docx]

**S1 File**

**Supplementary Information & Methods**

**Patient samples and ethics**

Human BM and peripheral blood (PB) specimens were residual material from appropriate clinical samples sent to the University of Birmingham immunophenotyping laboratory. Control samples were adult (ages 43-84yrs, median 64yrs) lymphoma patient staging marrows (n=24) with no evidence of haematological malignant cells (confirmed by morphology and flow cytometry), and umbilical cord blood (UCB) samples (n=4) obtained from the University of Birmingham Human Biomaterials Resource Centre. AML samples were residual material taken from AML adult patients (n=93, ages 16-69yrs, median 51yrs) for routine pre-treatment diagnostic analysis. 79 were de novo AML, 7 were secondary AML. Most patients were treated with standard daunorubicin chemotherapy for their first induction. For those patients with available clinical data complete remission (CR) was defined by morphology. *Flt3ITD* and *NPM1* mutational status was determined by PCR (as described in supplementary ref 1). BM samples from myelodysplastic syndrome (MDS)/myeloproliferative disease (MPD) patients were also studied (n=26, age 32-89yrs, median 76yrs). AML samples were between 24-36 hours old, due to time taken for delivery from different hospitals to our referral centre. All experiments with human clinical material were performed after receiving approval from the University of Birmingham Research Governance office and the North West - Greater Manchester East Research Ethics Committee (12/NW/0742) and were conducted according to the principles expressed in the Declaration of Helsinki.

**Immunophenotyping and ROS analysis of hematopoietic stem/progenitor cells**

White blood cells from control and leukemic/dysplastic BM/PB samples were isolated after red cell lysis with ammonium chloride solution. Cells were washed twice and then incubated with 1μM of 2‘- 7‘-dichlorofluorescein diacetate (Life Technologies), hereafter referred to as DCF, for 10min at 37°C 5% CO_2_. DCF-labelled cells were then washed and incubated with a cocktail of monoclonal antibodies (mAb) to allow gating of hematopoietic stem and progenitor cell (HSPC) subsets (see S1 Fig). Full details on mAb panels are shown in supplementary table 2. CD117-positive staining was chosen instead of lineage gating as CD117 is expressed by control HSC and other primitive myeloid precursor cells, as well as the overwhelming majority of AML and MDS stem/progenitor cells. This also allowed improved characterisation of CD34^+^ cells from CD34dim/low blast cells in AML specimens.

Staining for intracellular markers (ki67/BCL2) was performed after fixation and permeabilization of DCF and cell-surface marker labelled cells using a commercial fix/perm reagent (Life Technologies). Isotype control staining was performed for each sample subjected to intracellular staining. For cells subjected to intracellular staining, chloro-methyl DCF-DA (Life Technologies) was used due to its enhanced cytoplasmic retention after permeabilization. For assessment of cell viability, surface stained BM/PB cells were washed and then stained with Annexin-V and 7-aminoactinomycin D (7AAD) using an Annexin-V detection kit according to the manufacturer’s instructions (BD Biosciences). Drug sensitivity assays were performed after mononuclear cells were isolated from patient samples by Ficoll-based density gradient centrifugation. Data acquisition was performed on a BD-FACS-Canto-II flow cytometer. Between 500,000 to 1,000,000 total cells or a minimum of 5,000 blast cell events were acquired and recorded. Afterwards, the saved FCS files were analysed with FlowJo (v7.6) software. In ROS modification experiments, BM cells were treated overnight with the pro-oxidant BSO (Sigma-Aldrich UK) at final concentration of 100μM.

**Reference**

1. Lazenby M, Gilkes AF, Marrin C, Evans A, Hills RK and Burnett AK. The prognostic relevance of flt3 and npm1 mutations on older patients treated intensively or non-intensively: a study of 1312 patients in the UK NCRI AML16 trial. Leukemia 2014; 28(10):1953-9.
