# Supplementary material for: Normal Hematopoietic Progenitor Subsets Have Distinct Reactive Oxygen Species, BCL2 and Cell-Cycle Profiles That Are Decoupled from Maturation in Acute Myeloid Leukemia
Source: PLoS One. 2016 Sep 26;11(9):e0163291. doi: 10.1371/journal.pone.0163291 (PMC5036879; doi:10.1371/journal.pone.0163291)
Supplement: S1 Table — (DOCX) [file pone.0163291.s009.docx]

**S1 Table**

**AML patient / sample characteristics**

|  | **CD34^+^ AML (n=70)** | | | | | **CD34 ^̶^ AML (n=23)** |
| --- | --- | --- | --- | --- | --- | --- |
|  | **CD34^+^ SPC type** | | | | |  |
|  | LMPP/GMP-like  Total (BM/PB) | MPP/CMP-like  Total (BM/PB) | CMP/GMP-like  Total (BM/PB) | GMP-like  Total (BM/PB) | Mixed  Total (BM/PB) | Total (BM/PB) |
| de novo AML  secondary AML  unknown | 35 (14/21)  1 (1/0)  2 (0/2) | 3 (3/0)  2 (1/1)  2 (2/0) | 4 (2/2)  0 | 5 (1/4)  0 | 12 (4/8)  3 (1/2)  1 (0/1) | 20 (10/10)  1 (0/1)  2 (0/2) |
| CBF *  (favourable risk) | 9 (3/6) | 0 | 2 (1/1) | 0 | 1 (1/0) | 1 (1/0) |
| ***Flt3*-ITD/*NPM1* status** |  |  |  |  |  |  |
| ITD+*/NPM1*mut | 0 | 0 | 0 | 0 | 0 | 9 (4/5) |
| ITD+*/NPM1-*wt | 6 (1/5) | 0 | 0 | 0 | 0 | 0 |
| ITD-*/NPM1*mut | 3 (0/3) | 0 | 0 | 2 (1/1) | 2 (0/2) | 6 (2/4) |
| ITD-*/NPM1-*wt | 15 (9/6) | 5 (4/1) | 2 (1/1) | 3 (0/3) | 12 (4/8) | 5 (3/1) |
| Unknown | 5 (2/3) | 2 (2/0) | 0 | 0 | 1 (0/1) | 2 (0/2) |

* 11 of 13 CBF patients were *Flt3*-ITD*^-^*/*NPM1*wt, with mutation data unavailable on 2 patients.

LMPP/GMP indicates AML cases where blasts are >90% LMPP-/GMP-like

MPP/CMP indicates AML cases where blasts are >90% MPP-/CMP-like

Mixed indicates AMLs containing mixture of MPP/LMPP/CMP/GMP-like blasts, with no single subset exceeding 80% of blasts
