# Supplementary material for: Normal Hematopoietic Progenitor Subsets Have Distinct Reactive Oxygen Species, BCL2 and Cell-Cycle Profiles That Are Decoupled from Maturation in Acute Myeloid Leukemia
Source: PLoS One. 2016 Sep 26;11(9):e0163291. doi: 10.1371/journal.pone.0163291 (PMC5036879; doi:10.1371/journal.pone.0163291)
Supplement: S2 Table — (DOCX) [file pone.0163291.s010.docx]

**S2 Table**

**Monoclonal antibody and fluorochrome panels used in combined assays**

| **Tube** | | **FITC** | **PE** | **PerCP** | **Pe-Cy7** | **APC/ AF-647*** | **APC-H7** | **BV421/V450**** | **V500** |
| --- | --- | --- | --- | --- | --- | --- | --- | --- | --- |
| **HSPCs** | **1a** | DCF | CD123 | CD34 | CD117 | CD38 | CD45RA | CD90 | CD45 |
|  | **1b** | DCF | CD71 | CD34 | CD117 | CD38 | CD45RA | CD33/ CD235a | CD45 |
| **Cell-cycle** | **2a** | DCF | CD38 | CD34 | CD117 | ki67* | CD45RA | BCL2****** | CD45 |
|  | **2b** | CD38 | CD123 | CD34 | CD117 | ki67* | CD45RA | BCL2****** | CD45 |
| **Viability** | **3a** | Annexin V | CD123 | 7-AAD | CD117 | CD38 | CD45RA | CD34 | CD45 |
|  | **3b** | DCF | Annexin V | CD34 | CD117 | CD38 | CD45RA | CD123 | CD45 |

*anti-ki67 conjugated to Alexafluor-647 and ** anti-BCL2 conjugated to Horizon V450

Note - colour matched isotype controls for ki67 and BCL2 were used simultaneously in a separate tube

Tubes 1a, 2a, 3a was performed on all samples and additional tubes 1b, 2b, 3b on selected samples where sufficient material was available
